# Modulation of charge transfer by *N*-alkylation to control photoluminescence energy and quantum yield†

**DOI:** 10.1039/d0sc02460k

**Published:** 2020-06-09

**Authors:** Andrew T. Turley, Andrew Danos, Antonio Prlj, Andrew P. Monkman, Basile F. E. Curchod, Paul R. McGonigal, Marc K. Etherington

**Affiliations:** Department of Chemistry, Durham University South Road Durham DH1 3LE UK paul.mcgonigal@durham.ac.uk; Department of Physics, Durham University South Road Durham DH1 3LE UK marc.k.etherington@northumbria.ac.uk; Department of Mathematics, Physics and Electrical Engineering, Northumbria University Ellison Place NE1 8ST UK

## Abstract

Charge transfer in organic fluorophores is a fundamental photophysical process that can be either beneficial, *e.g.*, facilitating thermally activated delayed fluorescence, or detrimental, *e.g.*, mediating emission quenching. *N*-Alkylation is shown to provide straightforward synthetic control of the charge transfer, emission energy and quantum yield of amine chromophores. We demonstrate this concept using quinine as a model. *N*-Alkylation causes changes in its emission that mirror those caused by changes in pH (*i.e.*, protonation). Unlike protonation, however, alkylation of quinine's two N sites is performed in a stepwise manner to give kinetically stable species. This kinetic stability allows us to isolate and characterize an *N*-alkylated analogue of an ‘unnatural’ protonation state that is quaternized selectively at the less basic site, which is inaccessible using acid. These materials expose (i) the through-space charge-transfer excited state of quinine and (ii) the associated loss pathway, while (iii) developing a simple salt that outperforms quinine sulfate as a quantum yield standard. This *N*-alkylation approach can be applied broadly in the discovery of emissive materials by tuning charge-transfer states.

## Abstract 2

A versatile *N*-alkylation strategy controls the presence of charge-transfer excited states and the emission colour of N-heterocyclic chromophores.

## Graphical abstract

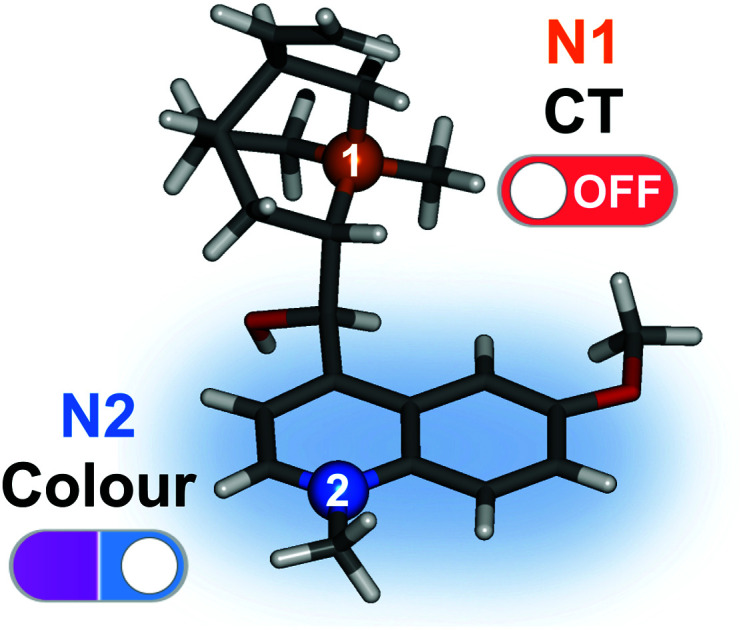

## Introduction

‘Your “epipolic” dispersion has given me the clue to a most extensive field of research, which has occupied me during the last year when sunlight permitted’ wrote Sir George Stokes to Sir John Herschel on April 6, 1852.^1^ This prediction of an extensive field of research has held true through the continued use of spectroscopy to study epipolic dispersion that, also thanks to Stokes, we now know as fluorescence. In recent years, fluorescence spectroscopy has been used to characterize a wide range of different compounds and uncover new functional phenomena such as thermally activated delayed fluorescence (TADF),^2–7^ aggregation induced emission^8–10^ and room temperature phosphorescence.^11–13^

TADF is of particular interest as it increases the efficiency of organic light-emitting diodes (OLEDs) used in displays and devices. The TADF process is contingent on the formation of charge-transfer (CT) states, *i.e.*, the spatial redistribution of electron density in the excited state. Controlling and tuning CT states is crucial to the development of high efficiency TADF materials.^14^ This redistribution can result in reduced overlap between the highest occupied molecular orbital (HOMO) and lowest unoccupied molecular orbital (LUMO).^4,15^ CT states also have a significant impact on photochemistry and are commonplace in natural chromophores – for example, they have been invoked in photosynthesis^16–18^ and DNA repair^19,20^ mechanisms. CT states can contribute to enhancing light emission in organic compounds through mechanisms such as TADF, but their presence is not always desirable. Identification of their behaviour with respect to the electronic energy levels can guide material design.

Here, we utilize quinine (**Qn**) as a model system to demonstrate a strategy for modifying CT states of organic compounds. Recent literature has shown that some of the cinchona alkaloids display CT and proton transfer, which in turn dictate their photophysical properties.^21,22^ These studies have previously been performed using protonation equilibria in aqueous media. Here, we use *N*-alkylation as an alternative in order to permanently modify **Qn**, allowing investigation into the structure–property relationships between lone-pair availability, charge, emission energy and quantum yield. We demonstrate that *N*-alkylation is a versatile method for studying the effects of amine quaternization in aqueous and organic solvent media, not requiring acidic conditions or being subject to equilibria. The kinetic stabilities of the *N*-alkylated salts also make it possible to quaternize structures at positions other than their most basic N site. We identify one N site of **Qn** that can be manipulated to ‘turn off’ CT state formation and another whose modification tunes emission colour. The compound produced by double *N*-alkylation displays enhanced photoluminescence quantum yields (PLQYs) and solubilities across a range of solvents compared to acidified quinine sulfate (**H2Qn**·SO_4_), which is a common PLQY standard for characterizing blue emitters. Overall, this *N*-alkylation approach represents a simple, robust pathway for tuning the emission and functional properties of **Qn** and other tertiary amines^23–26^ to impart functional properties such as improved PLQYs and, potentially, TADF emission.

## Results and discussion

**Scheme 1:**
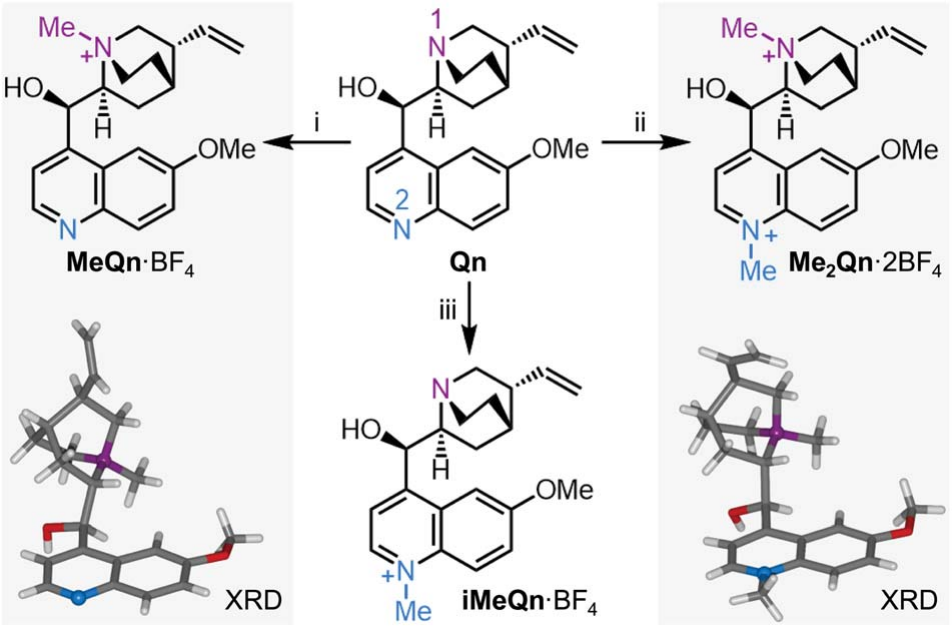
*N*-Alkylation of **Qn** to *N*-methylquinium tetrafluoroborate, **MeQn**·BF_4_, *N*,*N*′-dimethylquinium bis(tetrafluoroborate), **Me2Qn**·2BF_4_, and iso-*N*-methylquinium tetra-fluoroborate, **iMeQn**·BF_4_. XRD structures of **MeQn**·BF_4_ and **Me2Qn**·2BF_4_ are shown in stick representation with N atoms as balls. Further XRD structures can be found in Fig. S16, S17 and Table S1.† Reagents and conditions: (i) (a) MeI, rt, 3 d, (b) AgBF_4_, MeCN, 60 °C, 10 min, 74% over 2 steps; (ii) (a) MeI, MeCN, 100 °C, 4 h, (b) AgBF_4_, MeOH, rt, 10 min, 79% over 2 steps; (iii) (a) allyl bromide, CH_2_Cl_2_, rt, 16 h, (b) MeI, MeCN, 100 °C, 3 h, (c) barbituric acid, Pd(PPh_3_)_4_ (5 mol%), Me_2_SO, 40 °C, 16 h, (d) diisopropylaminomethyl polystyrene, MeOH, rt, 1 h, (e) AgBF_4_, MeOH, rt, 10 min, 79% over 5 steps.

**Qn** fluoresces blue with a high PLQY in acidified water (55% in 0.1 M aqueous H_2_SO_4_), but is only weakly emissive at UV wavelengths in basic solution.^27^ Recent work has shown that a similar pH-controlled increase in the PLQY of dehydroquinidine, an analogue of **Qn**, is linked to the availability of the quinuclidine lone pair electrons.^21^ It has been proposed that formation of a through-space CT state between the quinuclidine N and the quinoline chromophore leads to quenching of emission. This CT loss pathway is suppressed when the quinuclidine N is protonated, which increases the PLQY. We reasoned that *N*-alkylation of **Qn** could modulate its CT state in a similar manner and that investigation of *N*-alkylated derivatives would allow us to delineate the separate effects of quaternizing each of the two N sites (N1 and N2, Scheme 1) independently of one another, circumventing the intrinsic limitations of using protonation for this task.

We synthesized three salts of methylated **Qn** (Scheme 1). Synthetic procedures and NMR spectroscopic characterisation can be found in Scheme S1 and Fig. S1–S13 in the ESI.† Selective *N*-methylation of the quinuclidine N (N1) is achieved by treating **Qn** with MeI at room temperature,^28^ giving **MeQn**^+^, whereas a reaction temperature of 100 °C leads to **Me2Qn**^2+^ by *N*-methylation of both N1 and the quinoline N (N2).^29,30^ This selectivity follows the known basicity trend of N1 and N2.^21,31,32^ Methylation at each site gives characteristic changes in ^1^H NMR chemical shifts (Fig. S14 and S15†) and is confirmed by X-ray diffraction (XRD) analysis of single crystals (Scheme 1).† By employing an allyl protecting group at N1 (Scheme S1†), however, it is also possible to prepare an isomeric form of **MeQn**^+^ that would be inaccessible using a thermodynamically controlled quaternization approach, such as reversible protonation under acidic conditions. After methylation at N2, the allyl protecting group at N1 is removed to give the kinetically stable ion **iMeQn**^+^. Each of the cations were isolated with halide counterions before exchanging to BF_4_ salts by metathesis with AgBF_4_ to avoid heavy-nucleus ions with large spin–orbit couplings that might complicate our photophysical investigations by quenching the singlet emission.^33^

**Fig. 1:**
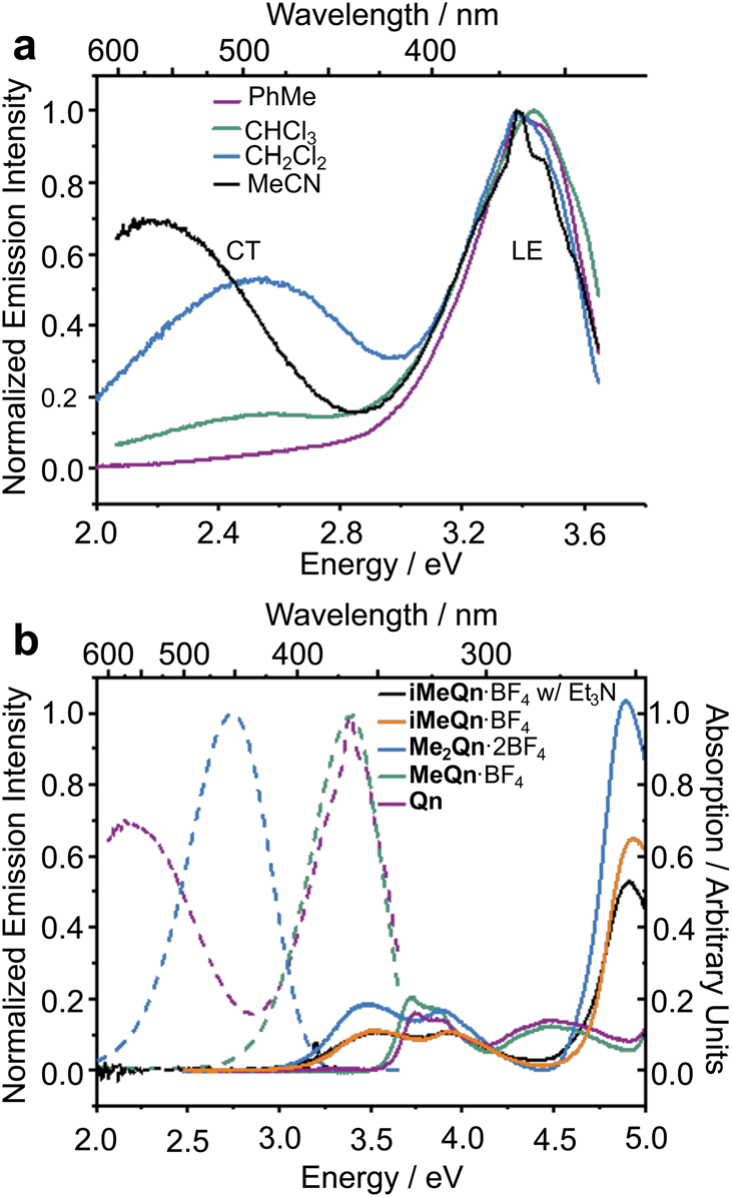
(a) Steady-state emission spectra of **Qn** dissolved in a series of solvents and excited at 3.75 eV, showing the LE and CT character of its emission. (b) The absorption (solid lines) and emission (dashed lines) spectra of **Qn** (purple), **MeQn**·BF_4_ (green), **Me2Qn**·2BF_4_ (blue), and **iMeQn**·BF_4_ (orange) in MeCN (20 μM). A significant shift in the absorption spectra is associated with the methylation of N2. The emission spectrum of **iMeQn**·BF_4_ in MeCN is omitted as partial protonation of N1 by adventitious water affects the spectrum. Instead, the absorption and emission spectra (black) of a MeCN solution of **iMeQn**·BF_4_ (20 μM) with Et_3_N (10 mM) are displayed to show there is no significant change in absorption with the addition of a base and that there is no emission in the visible region without protonation of N1. The excitation energies (*E*_ex_) used were 4.13 eV for **Qn** and **MeQn**·BF_4_ or 3.54 eV for **Me2Qn**·2BF_4_ and **iMeQn**·BF_4_ to allow comparison of PLQYs with known standards (Fig. S18–S26 and Table S2†).

The photophysical properties we have measured and modelled for **Qn** and its salts are summarized in Table 1.^34^ We first recorded (Fig. 1a) emission spectra of **Qn** dissolved in a series of solvents with a range of polarities. Although the absolute emission intensities from **Qn** in polar organic solvents are low on account of its near-zero PLQY, we could identify distinct emissions from a locally excited (LE) and a CT state.

**Table 1:** Photophysical properties of **Qn** and its methylated derivatives

Compound	*Φ* a/%		*E*_em_/eV		Calculated *E*_em_^d^/eV		Presence of CT^e^	Red-shifted absorption and emission^f^
	MeCN^b^	H_2_O	MeCN^b^	H_2_O	LE	CT		
**Qn**	0	22	3.45 (2.30)	3.20	3.75	1.41	Yes	No
**MeQn**·BF_4_	5	32	3.40	3.24	3.69	—	No	No
**Me2Qn**·2BF_4_	63	70	2.75	2.75	3.00	—	No	Yes
**iMeQn**·BF_4_	0^c^	60	—	2.75	—	1.01	Yes	Yes

a PLQYs (*Φ*) for **Qn** and **MeQn**·BF_4_ were measured with respect to a standard of 2-aminopyridine in 0.1 M aqueous H_2_SO_4_ (*Φ* = 60%)^38^ and those of **Me2Qn**·2BF_4_ and **iMeQn**·BF_4_ were measured with respect to a standard of **H2Qn**·SO_4_ in 0.1 M aqueous H_2_SO_4_ (*Φ* = 55%).^27^

b Anhydrous MeCN was used throughout the spectroscopic study.

c Anhydrous MeCN solution with 10 mM Et_3_N used to suppress the formation of trace amounts of N1 protonated species resulting from adventitious water.

d Excited-state energies in eV for protonated (rather than methylated) compounds calculated at the LR-TDDFT/ωB97X-D/6-31G* level of theory with state-specific implicit solvation (MeCN).

e The presence or lack of an accessible CT state was determined by experimental observation (Fig. 1) and/or theoretical calculation (Tables S5 and S6).

f Relative to the absorption and emission of **Qn**.

**Fig. 2:**
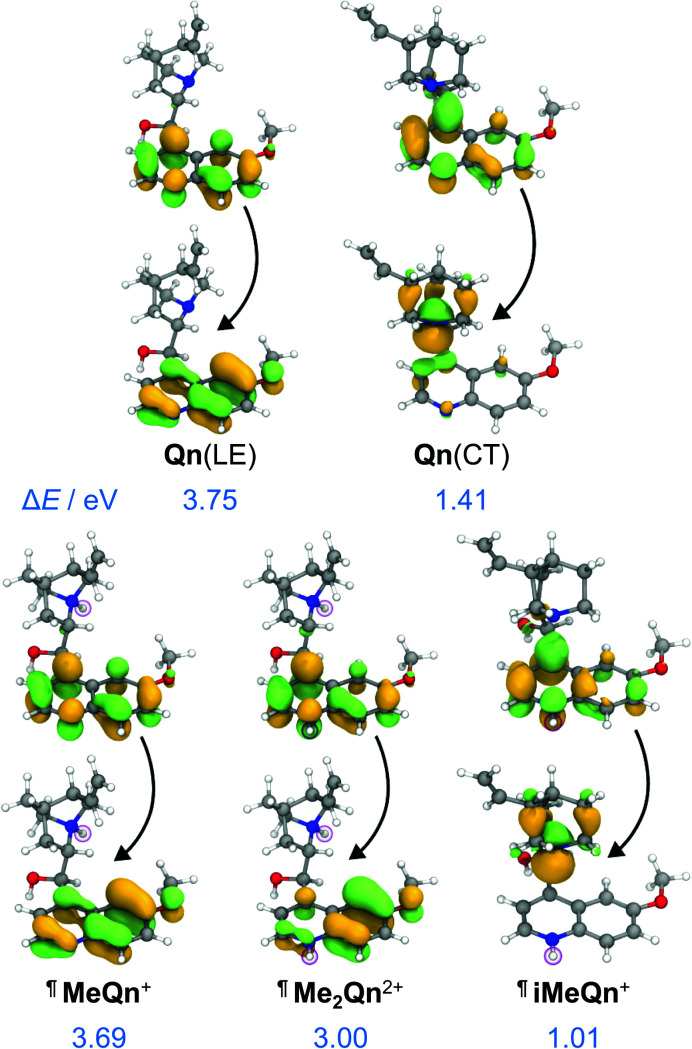
Natural transition orbitals (NTOs) and energies characterizing the singlet emission of **Qn** from its LE and CT states, as well as **MeQn**^+^, **iMeQn**^+^ and **Me2Qn**^2+^, calculated at the LR-TDDFT/ωB97X-D/6-31G* level of theory with state-specific implicit solvation. ¶ indicates that protonated structures were used as electronically similar (Table S7†) models for the methylated salts.

A peak in the UV region with emission energy, *E*_em_, of 3.4 eV is present in low polarity solvent (PhMe), consistent with emission from an LE state. An additional peak is present in the visible region between 2.2 and 2.6 eV in more polar media. As the polarity is increased when moving from chlorinated solvents to MeCN, the increasing relative intensity of this second peak and its further bathochromic shift are indicative of emission from a CT state.^35–37^ The emission spectra are normalized relative to the peak of the LE (which has reduced intensity in polar solvents) in order to better demonstrate the solvatochromic shift. The intensities of the spectra alone do not illustrate the absolute populations of the states, but the changes in intensity can be rationalized. In apolar solvents there is a higher population of molecules emitting from the LE state, having a high oscillator strength. In polar solvents an important population of molecules is now emitting from the CT state, but the low oscillator strength results in a similar magnitude of emission relative to the LE state. Linear-response time-dependent density functional theory (LR-TDDFT) calculations performed at the ωB97X-D/6-31G* level of theory with state-specific implicit solvation (see ESI† for full all computational details and benchmarking) support the assignment of these LE and CT bands of **Qn** (Fig. 2). The CT state, in this case, is a result of a through-space charge transfer between the quinuclidine and quinoline system.

The calculations predict (i) UV emission associated with an electronic transition localized on the quinoline ring system (π → π*) and (ii) a lower energy emission from a CT excited state, which is associated with an electronic transition from a nonbonding orbital located on N1 to an unoccupied π orbital on the quinoline ring (n → π*). We ascribe **Qn**'s extremely low PLQY (Table 1) to the formation of this through-space CT state. Rapid nonradiative decay from the low-energy CT state, possibly through the low-lying LE triplet state (Fig. S27†), serves as a loss pathway that suppresses photoluminescence.^39^

To further confirm this hypothesis and to ‘switch on’ the UV emission of **Qn**, we investigated the effect of alkylating N1 selectively. Pleasingly, we observed (Fig. 1b) only a single peak in the emission spectrum of **MeQn**·BF_4_ at 3.4 eV in MeCN, indicative of emission from the π → π* LE state of the quinoline ring system. Compared to **Qn**, the peak wavelengths observed in the absorption and emission spectra of **MeQn**·BF_4_ are largely unchanged. However, **MeQn**·BF_4_ shows no evidence of the lower energy emission from a CT state found for **Qn**. Consequently, **MeQn**·BF_4_ emits (Table 1) with an increased PLQY of 5% in MeCN, compared to the near-zero PLQY of neutral **Qn** in MeCN. Quaternization at N1 prevents formation of a CT excited state, removing one of the pathways for rapid nonradiative decay and, as a result, enhancing the PLQY. Similarly, CT state formation is also suppressed for neutral H_2_O solutions of **Qn**, in which the major species at equilibrium is monoprotonated **HQn+**,^31^ giving a PLQY of 22%. The PLQY of **MeQn**·BF_4_ in H_2_O solution is 32%.

Methylation of N2 has a markedly different effect on the photoluminescence properties. We first investigated this effect by quaternizing both N1 and N2. The doubly methylated salt **Me2Qn**·2BF_4_ exhibits (Fig. 1b) significant bathochromic shifts in its absorption and emission energies relative to **Qn** and **MeQn**·BF_4_. These shifts are reproduced qualitatively by our LR-TDDFT (Table 1) and algebraic diagrammatic construction (ADC(2)) calculations (Tables S3 and S4†). Like **MeQn**·BF_4_, emission from **Me2Qn**·2BF_4_ (Fig. 2) comes exclusively from the π → π* LE state. However, the transition is brought into the blue region (2.8 eV) as a result of quaternizing N2.

Extrapolating from our observations based on **MeQn**·BF_4_ and **Me2Qn**·2BF_4_, we would expect that selective quaternization of only N2—the less basic N site—would give rise to an ion that retains the CT character of **Qn** on account of the available quinuclidine lone pair electrons (N1), but whose emission and absorption are red-shifted. The LR-TDDFT calculations fit with this hypothesis, predicting (Fig. 2) formation of a new low-energy CT state at lower electronic energy. Moreover, the LR-TDDFT indicates that, upon relaxation of the excited state, the CT state becomes the primary (singlet) ‘emissive’ state. The selectivity and kinetic stability of the *N*-alkylation approach allows us to test this hypothesis experimentally using **iMeQn**·BF_4_.

As predicted, the absorption spectrum of **iMeQn**·BF_4_ in MeCN matches closely (Fig. 1b) the spectrum of **Me2Qn**·2BF_4_. Photoluminescence measurements, which were carried out in MeCN with 10 mM triethylamine (Et_3_N) to prevent protonation of N1 by adventitious water, are also consistent. Unlike the other methylated derivatives, there is no detectable photoluminescence from **iMeQn**·BF_4_. Population of the extremely low-energy CT excited state predicted by calculation appears to lead solely to non-radiative decay. Overall, therefore, selective alkylation of different sites can be used to tune the photophysical behaviour of **Qn**, modulating independent properties in an orthogonal manner. It is a useful approach to control CT and emission energy. However, the kinetic stabilities and modified solubility profiles of the **Qn** salts also give them advantages over protonated analogues for use as functional materials. For example, while a doubly protonated **Qn** salt, **H2Qn**·SO_4_, is used routinely as a fluorescence standard for relative PLQY measurements in the blue region,^10^ its use is limited to acidic aqueous solutions – it is insoluble in common organic solvents and its photoluminescence is quenched in the aggregated state. The physical properties of **Me2Qn**·2BF_4_, on the other hand, make it appealing as a more versatile PLQY standard.

Our measurements show that it exhibits an enhanced PLQY of 70% in neutral H_2_O compared to the 55% PLQY of **H2Qn**·SO_4_ in 0.1 M aqueous H_2_SO_4_ (ref. 27) and, unlike **H2Qn**·SO_4_, it is soluble in organic solvents such as MeCN, EtOH, Me_2_CO, CH_2_Cl_2_, and EtOAc, giving rise to useful PLQYs (Fig. S18–S26 and Table S2†) of 63%, 43%, 54%, 48% and 13%, respectively. The variation in PLQY may be a result of modulating ion-pairing between the fluorescent cation and its counterion. Previous investigations of cationic fluorophores have also shown solvent-dependent PLQY and have suggested interactions with the counterion can provide radiationless decay pathways, *e.g.*, through electron transfer and heavy atom effects.^40,41^ Overall, however, simple counterion exchange can be used to tune the solubility profile and emission properties. Work is ongoing in our laboratories to optimize these materials as PLQY standards and provide physical insight into the empirical changes in PLQY with solvent.

## Conclusions

In summary, we have demonstrated an operationally simple method to tune and elucidate the CT excited states of emissive organic materials by *N*-alkylation. In the present investigation, this approach has allowed us to systematically toggle on and off the presence of a CT excited state, to change the emission colour, and to improve the PLQY of **Qn** – a compound whose photophysical properties were first studied by Herschel and Stokes over 150 years ago.^42–44^ This approach can be applied broadly to N-heterocycles, which are pervasive structural motifs in many organic chromophores. Resulting insights into their CT excited states will progress our understanding of natural photoactive systems^16–18,45^ and improve the performances of emissive^2–7,39^ and light-absorbing^46–48^ devices. The kinetic stabilities of the *N*-alkylated compounds open up the possibility of quaternizing the chromophores selectively at sites other than their most basic N site, achieving structures that are inaccessible by protonation, while the choice of counterion can serve as a handle to control physical properties.

## Conflicts of interest

There are no conflicts to declare.

## Supplementary Material

SC-011-D0SC02460K-s001

SC-011-D0SC02460K-s002

## References

[cit1] Stokes G. G., Sir George Gabriel Stokes to Sir John Frederick William Herschel, 6th April 1852, The Royal Society Archive, HS/17/27, 1852

[cit2] Uoyama H., Goushi K., Shizu K., Nomura H., Adachi C. (2012). Nature.

[cit3] Etherington M. K., Gibson J., Higginbotham H. F., Penfold T. J., Monkman A. P. (2016). Nat. Commun..

[cit4] Dias F. B., Bourdakos K. N., Jankus V., Moss K. C., Kamtekar K. T., Bhalla V., Santos J., Bryce M. R., Monkman A. P. (2013). Adv. Mater..

[cit5] Stachelek P., Ward J. S., dos Santos P. L., Danos A., Colella M., Haase N., Raynes S. J., Batsanov A. S., Bryce M. R., Monkman A. P. (2019). ACS Appl. Mater. Interfaces.

[cit6] Congrave D. G., Drummond B. H., Conaghan P. J., Francis H., Jones S. T. E., Grey C. P., Greenham N. C., Credgington D., Bronstein H. (2019). J. Am. Chem. Soc..

[cit7] Izumi S., Higginbotham H. F., Nyga A., Stachelek P., Tohnai N., De Silva P., Data P., Takeda Y., Minakata S. (2020). J. Am. Chem. Soc..

[cit8] Mei J., Leung N. L. C., Kwok R. T. K., Lam J. W. Y., Tang B. Z. (2015). Chem. Rev..

[cit9] Sturala J., Etherington M. K., Bismillah A. N., Higginbotham H. F., Trewby W., Aguilar J. A., Bromley E. H. C., Avestro A.-J., Monkman A. P., McGonigal P. R. (2017). J. Am. Chem. Soc..

[cit10] Zhang H., Zhao Z., McGonigal P. R., Ye R., Liu S., Lam J. W. Y., Kwok R. T. K., Yuan W. Z., Xie J., Rogach A. L., Tang B. Z. (2020). Mater. Today.

[cit11] Pander P., Swist A., Turczyn R., Pouget S., Djurado D., Lazauskas A., Pashazadeh R., Grazulevicius J. V., Motyka R., Klimash A., Skabara P. J., Data P., Soloducho J., Dias F. B. (2018). J. Phys. Chem. C.

[cit12] Kukhta N. A., Huang R., Batsanov A. S., Bryce M. R., Dias F. B. (2019). J. Phys. Chem. C.

[cit13] Salla C. A. M., Farias G., Rouzières M., Dechambenoit P., Durola F., Bock H., de Souza B., Bechtold I. H. (2019). Angew. Chem. Int. Ed..

[cit14] Huang R., Kukhta N. A., Ward J. S., Danos A., Batsanov A. S., Bryce M. R., Dias F. B. (2019). J. Mater. Chem. C.

[cit15] dos Santos P. L., Etherington M. K., Monkman A. P. (2018). J. Mater. Chem. C.

[cit16] Meech S. R., Hoff A. J., Wiersma D. A. (1986). Proc. Natl. Acad. Sci. U. S. A..

[cit17] Novoderezhkin V. I., Dekker J. P., van Grondelle R. (2007). Biophys. J..

[cit18] Ahn T. K., Avenson T. J., Ballottari M., Cheng Y.-C., Niyogi K. K., Bassi R., Fleming G. R. (2008). Science.

[cit19] Bucher D. B., Kufner C. L., Schlueter A., Carell T., Zinth W. (2016). J. Am. Chem. Soc..

[cit20] Martinez-Fernandez L., Zhang Y., de La Harpe K., Beckstead A. A., Kohler B., Improta R. (2016). Phys. Chem. Chem. Phys..

[cit21] Qin W., Vozza A., Brouwer A. M. (2009). J. Phys. Chem. C.

[cit22] Qian J., Brouwer A. M. (2010). Phys. Chem. Chem. Phys..

[cit23] Wang K.-L., Liou W.-T., Liaw D.-J., Huang S.-T. (2008). Polymer.

[cit24] Zhang J., Chen J., Xu B., Wang L., Ma S., Dong Y., Li B., Ye L., Tian W. (2013). Chem. Commun..

[cit25] Achelle S., Rodríguez-Lopez J., Katan C., Robin-Le Guen F. (2016). J. Phys. Chem. C.

[cit26] Matos M. J., Navo C. D., Hakala T., Ferhati X., Guerreiro A., Hartmann D., Bernardim B., Saar K. L., Compañón I., Corzana F., Knowles T. P. J., Jiménez-Osés G., Bernardes G. J. L. (2019). Angew. Chem. Int. Ed..

[cit27] Melhuish W. H. (1961). J. Phys. Chem..

[cit28] McNeice P., Vallana F. M. F., Coles S. J., Horton P. N., Marr P. C., Seddon K. R., Marr A. C. (2020). J. Mol. Liq..

[cit29] Hesse O. (1885). Justus Liebigs Ann. Chem..

[cit30] Yadav A. K., Singh A. (2000). Synlett.

[cit31] Schulman S. G., Threatte R. M., Capomacchia A. C., Paul W. L. (1974). J. Pharm. Sci..

[cit32] Xiang B., Belyk K. M., Reamer R. A., Yasuda N. (2014). Angew. Chem. Int. Ed..

[cit33] Gutow J. H. (2005). J. Chem. Educ..

[cit34] Mooney J., Kambhampati P. (2013). J. Phys. Chem. Lett..

[cit35] Brunschwig B. S., Ehrenson S., Sutin N. (1987). J. Phys. Chem..

[cit36] Verbeek G., Depaemelaere S., Van der Auweraer M., De Schryver F. C., Vaes A., Terrell D., De Meutter S. (1993). Chem. Phys..

[cit37] Laguitton-Pasquier H., Pansu R., Chauvet J.-P., Collet A., Faure J., Lapouyade R. (1996). Chem. Phys..

[cit38] Rusakowicz R., Testa A. C. (1968). J. Phys. Chem..

[cit39] Etherington M. K., Franchello F., Gibson J., Northey T., Santos J., Ward J. S., Higginbotham H. F., Data P., Kurowska A., Dos Santos P. L., Graves D. R., Batsanov A. S., Dias F. B., Bryce M. R., Penfold T. J., Monkman A. P. (2017). Nat. Commun..

[cit40] Avilov I. V., Panarin A. Y., Chirvony V. S. (2004). Chem. Phys. Lett..

[cit41] Knyukshto V. N., Solovyov K. N., Egorova G. D. (1998). Biospectroscopy.

[cit42] Herschel J. F. W. (1845). Philos. Trans. R. Soc. London.

[cit43] Stokes G. G. (1852). Philos. Trans. R. Soc. London.

[cit44] Valeur B., Berberan-Santos M. N. (2011). J. Chem. Educ..

[cit45] Bai S., Balevicius V., Bittner E., Cheng Y. C., Chergui M., Cina J., Das Neves Rodrigues N., Datta A., Dawlaty J., Dodin A., Fingerhut B., Fleming G., Ginsberg N., Hammes-Schiffer S., Huxter V., Kohler B., Lee Y., Leggett G., Marcus A., Morenz K., Ogilvie J., Olaya-Castro A., Oliver T. A. A., Son M., Song Y., Stavros V. (2019). Faraday Discuss..

[cit46] Rao A., Chow P. C. Y., Gélinas S., Schlenker C. W., Li C., Yip H., Jen A. K.-Y., Ginger D. S., Friend R. H. (2013). Nature.

[cit47] Etherington M. K., Wang J., Chow P. C. Y., Greenham N. C. (2014). Appl. Phys. Lett..

[cit48] Chang W., Congreve D. N., Hontz E., Bahlke M. E., McMahon D. P., Reineke S., Wu T. C., Bulović V., Van Voorhis T., Baldo M. A. (2015). Nat. Commun..

