# Supplementary material for: Modulation of charge transfer by N-alkylation to control photoluminescence energy and quantum yield
Source: Chem Sci. 2020 Jun 9;11(27):6990–5. doi: 10.1039/d0sc02460k (PMC8159361; doi:10.1039/d0sc02460k)
Supplement: SC-011-D0SC02460K-s001 [file SC-011-D0SC02460K-s001.pdf]

Electronic Supplementary Material (ESI) for Chemical Science.
This journal is © The Royal Society of Chemistry 2020

Modulation of Charge Transfer by *N*-Alkylation to Control Fluorescence Energy and Quantum Yield

Andrew T. Turley[a], Andrew Danos[b], Antonio Prlj[a], Andrew P. Monkman[b], Basile F. E. Curchod[a], Paul R. McGonigal[a,*], Marc K. Etherington[b,c*]

[a] Department of Chemistry and [b] Department of Physics, Durham University, Lower Mountjoy, Stockton Road, Durham, DH1 3LE, UK. [c] Department of Mathematics, Physics and Electrical Engineering, Northumbria University, Ellison Place, Newcastle upon Tyne, NE1 8ST, UK.

Table of Contents

1. General Methods S1
2. Synthetic Procedures S3
3. ^{1}H, ^{13}C and ^{19}F NMR Spectroscopic Characterisation of Synthesised Compounds S8
4. X-Ray Crystallographic Analysis S23
    4.1. **MeQn**·BF_4 S23
    4.2. **Me_2Qn**·$2BF_4$ S24
5. Photoluminescence Quantum Yield Measurements S26
6. Phosphorescence of **Qn** in a Zeonex Film S36
7. Theoretical Study S37
8. References S43

Supporting Information

1. General Methods

Materials: All reagents were purchased from commercial suppliers (Sigma-Aldrich, Acros Organics, or Alfa Aesar) and used without further purification.

Instrumentation and Analytical Techniques: Analytical thin-layer chromatography (TLC) was performed on neutral aluminium-sheet silica gel plates and visualised under UV irradiation (254 nm). Nuclear magnetic resonance (NMR) spectra were recorded using a Bruker Advance (III)-400 (^{1}H 400.130 MHz and ^{13}C 100.613 MHz), Varian Inova-500 (^{1}H 500.130 MHz and ^{13}C 125.758 MHz), Varian VNMRS-600 (^{1}H 600.130 MHz and ^{13}C 150.903 MHz) or a Varian VNMRS-700 (^{1}H 700.130 MHz and ^{13}C 176.048 MHz) spectrometers, at a constant temperature of 298 K unless otherwise stated. Chemical shifts (δ) are reported in parts per million (ppm) relative to the signals corresponding to residual non-deuterated solvents [DMSO-*d6*: δ = 2.50 or 39.52. CD_3OD: δ = 3.31 or 49.00]. Coupling constants (*J*) are reported in Hertz (Hz). ^{13}C NMR Experiments were proton-decoupled, whereas ^{19}F NMR experiments are coupled and referenced to an internal standard, hexafluorobenzene (HFB, δ = 164.99 ppm). Assignments of ^{1}H and ^{13}C NMR signals were accomplished by two-dimensional NMR spectroscopy (COSY, NOESY, HSQC, HMBC). NMR spectra were processed using MestReNova version 12. Data are reported as follows: chemical shift; multiplicity; coupling constants; integral and assignment. High-resolution electrospray (HR-ESI) mass spectra were measured using a Waters LCT Premier XE high resolution, accurate mass UPLC ES MS. Melting points were recorded using a Gallenkamp (Sanyo) apparatus and are uncorrected. UV-Vis-NIR absorbance spectra of solution samples were recorded using an Agilent Technologies Cary Series UV-vis-NIR spectrophotometer at room temperature. Steady-state photoluminescence of films and solutions were measured using a Jobin Yvon Fluoromax or Fluorolog with machine-specific calibration curves. The photoluminescence quantum yields (PLQYs) of the compounds were

measured using the relative method[1] and comparing to known standards of quinine sulfate[2] and 2-aminopyridine[3] in aqueous 0.1 M H_2SO_4. The absorption spectra and photoluminescence spectra for the PLQYs were measured on a Shimadzu UV-3600 UV-VIS-NIR spectrophotometer and a Jobin Yvon Fluoromax or Fluorolog. The low temperature phosphorescence spectrum of **Qn** in a zeonex film was acquired by placing the sample in a Janis Research VNF-100 cryostat, used in conjunction with a Lakeshore 332 temperature controller, and exciting the sample with 337nm light from an N_2 laser (LTBMNL 100, Lasertechnik Berlin) at 10 Hz. Sample emission was the directed onto a spectrograph and gated iCCD camera (Stanford Computer Optics). The X-ray single crystal data were collected at a temperature of 120.0(2) K using λCuKα radiation (λ = 1.54178 Å) on a Bruker D8Venture (Photon100 CMOS detector, IμS-microsource, focusing mirrors) diffractometer equipped with a Cryostream (Oxford Cryosystems) open-flow nitrogen cryostat. Both structures were solved by direct method and refined by full-matrix least squares on F^2 for all data using Olex2[4] and SHELXTL[5] software. All non-disordered non-hydrogen atoms were refined anisotropically, closely located disordered atoms in structure **MeQn**·BF_4 were refined isotropically. The disordered atoms were refined with fixed site occupation factors 0.6 and 0.4. Hydrogen atoms in structure **Me₂Qn**·2BF_4 and in OH-groups of structure **MeQn**·BF_4 were refined isotropically, the remaining hydrogen atoms in structure **MeQn**·BF_4 were placed in the calculated positions and refined in riding mode. The absolute configuration of studied compounds was determined from anomalous scattering by calculating the Flack[6] (*x*) and Hooft[7] (*y*) parameters which should equal 0 for the correct absolute structure and 1 for the inverted model. Crystal data and parameters of refinement are listed in Table S1. Crystallographic data for the structure have been deposited with the Cambridge Crystallographic Data Centre as supplementary publication CCDC-1985987-1985988.

2. Synthetic Procedures

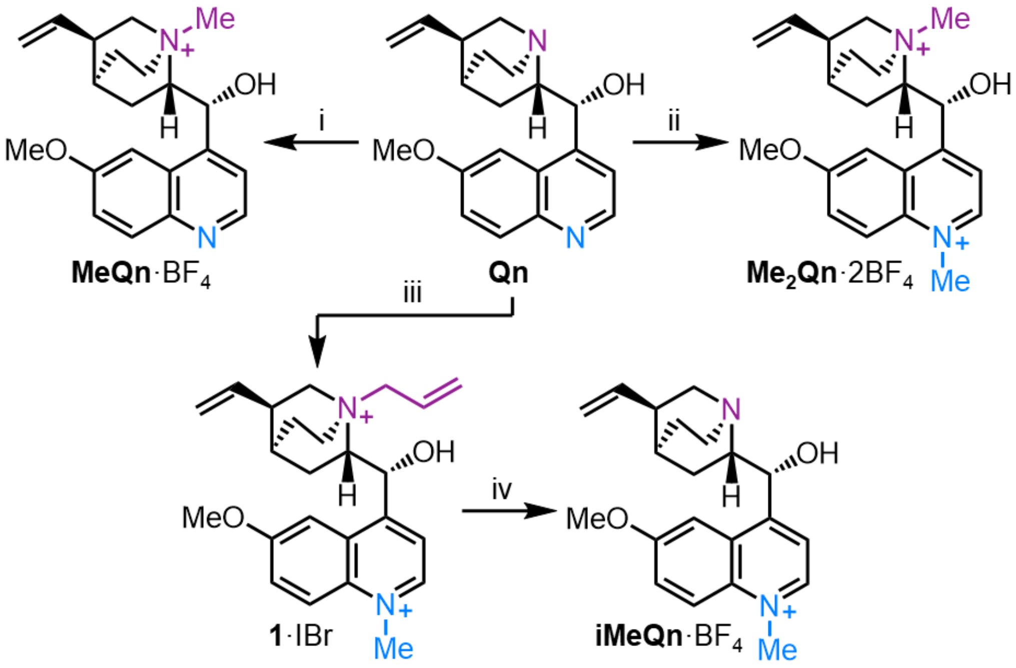

Scheme S1 Synthetic routes to **MeQn**·BF_4, **Me$_2$Qn**·BF_4 and **iMeQn**·BF_4. Reagents and conditions: (i) a) MeI / rt / 3 d, b) $AgBF_4$ / MeCN / 60 °C / 10 min; (ii) a) MeI / MeCN / 100 °C / 4 h, b) $AgBF_4$ / MeOH / rt / 10 min, (iii) a) Allyl bromide / CH_2Cl_2 / rt / 16 h, b) MeI / MeCN / 100 °C / 3 h; (iv) (a) Barbituric acid / $Pd(PPh_3)_4$ (5 mol%) / Me_2SO / 40 °C / 16 h, (b) Diisopropylaminomethyl polystyrene / MeOH / rt / 1h, c) $AgBF_4$ / MeOH / rt / 10 min.

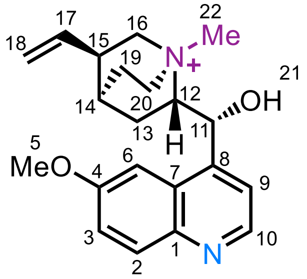

MeQn·BF_4: Quinine (500 mg, 1.54 mmol) was added to an oven-dried microwave vial and sealed. MeI (5 mL) was added and the mixture was sonicated at rt for 20 min. The reaction mixture was stirred for 3 days at rt. The mixture was filtered to isolate the precipitate, washing with CH_2Cl_2 (2 × 5 mL) and drying the precipitate under high vacuum. The resulting solid was recrystallized from MeCN to yield the iodide salt of title compound, **MeQn**·I, as a crystalline solid (618 mg, 1.23 mmol, 80 %). **MeQn**·I (106 mg, 0.227 mmol) was dissolved in MeCN (8 mL) and heated to 60 °C. A solution of $AgBF_4$ (44.0 mg, 0.227 mmol) in MeCN (1 mL) was added dropwise and allowed to stir at 60 °C

for 10 min. The resulting mixture was filtered and the filtrate was evaporated to dryness to give the title compound as a colourless solid (87 mg, 0.20 mmol, 93%). **M.P.** 185 – 187 °C. **^{1}H NMR** (700 MHz, DMSO-*d*$_6$) δ 8.79 (d, *J* = 4.5 Hz, 1H, H_{10}), 8.00 (d, *J* = 9.2 Hz, 1H, H_2), 7.70 (d, *J* = 4.9 Hz, 1H, H_9), 7.48 (dd, *J* = 9.2, 2.7 Hz, 1H, H_3), 7.21 (d, *J* = 2.7 Hz, 1H, H_6), 6.54 (d, *J* = 2.7 Hz, 1H, H_{21}), 6.21 (d, *J* = 3.2 Hz, 1H, H_{11}), 5.75 (ddd, *J* = 17.3, 10.5, 6.8 Hz, 1H, H_{17}), 5.13 – 5.01 (m, 2H, H_{18}), 4.07 (m, 1H, H_{20}), 4.00 (s, 3H, H_5), 3.74 – 3.60 (m, 3H, H_{16+12}), 3.45 – 3.35 (m, 4H, H_{20+22}), 2.85 – 2.76 (m, 1H, H_{15}), 2.20 – 2.10 (m, 2H, H_{13+19}), 2.06 – 2.01 (m, 1H, H_{14}), 1.97 – 1.87 (m, 1H, H_{19}), 1.38 – 1.31 (m, 1H, H_{13}). **^{13}C NMR** (176 MHz, DMSO-*d*$_6$) δ 157.4 (C_4), 147.4 (C_{10}), 143.8 (C_8), 143.6 (C_1), 137.9 (C_{17}), 131.5 (C_2), 125.1 (C_7), 121.5 (C_3), 119.9 (C_9), 116.5 (C_{18}), 101.6 (C_6), 66.78 (C_{12}), 63.9 (C_{11} + C_{16}), 55.48 (C_5), 54.2 (C_{20}), 48.7 (C_{22}), 37.6 (C_{15}), 25.9 (C_{14}), 24.6 (C_{19}), 19.3 (C_{13}). **^{19}F NMR** (376 MHz, CD_3OD) δ -150.48 (m, ^{10}B), -150.54 (m, ^{11}B). **HR-ESI-MS** *m/z* = 339.2088 $[M\text{-}BF_4]^+$ (calculated for $C_{21}H_{27}N_2O_2$ = 339.2073).

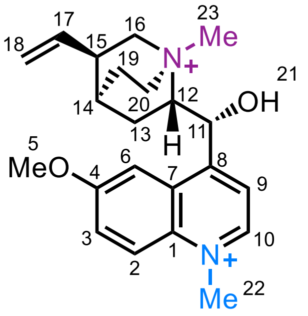

Me$_2$Qn·$2BF_4$: Quinine (2.00 g, 6.17 mmol) was added to an oven-dried microwave vial and sealed. MeCN (18 mL) was added and stirred at rt before adding MeI (1.92 mL, 30.8 mmol). The resulting mixture was heated at 100 °C for 4 h then allowed to cool to rt and further cooled to 5 °C in a fridge to allow the product to crystallise. The supernatant was decanted and the resulting crystals were isolated and dried under high vacuum to give the diiodide salt of the title compound (2.98 g, 4.9 mmol, 79%). **Me$_2$Qn**·2I (200 mg, 0.329 mmol) was dissolved in MeOH (5 mL) and a solution of $AgBF_4$ (128 mg, 0.658 mmol) in MeOH (5 mL) was added dropwise and stirred at rt for 10 min. The reaction mixture was filtered and the filtrate was evaporated to dryness to yield the

title compound as a colourless solid (135 mg, 0.329 mmol, quantitative). **M.P.** 195 – 197 °C. **^{1}H NMR** (600 MHz, DMSO-d_6) δ 9.32 (d, J = 6.1 Hz, 1H, H_{10}), 8.51 (d, J = 9.7 Hz, 1H, H_2), 8.28 (d, J = 6.0 Hz, 1H, H_9), 7.99 (dd, J = 9.7, 2.6 Hz, 1H, H_3), 7.54 (d, J = 2.7 Hz, 1H, H_6), 7.16 – 7.05 (m, 1H, H_{21}), 6.53 – 6.46 (m, 1H, H_{11}), 5.70 (ddd, J = 17.3, 10.5, 6.9 Hz, 1H, H_{17}), 5.13 – 5.03 (m, 2H, H_{18}), 4.62 (s, 3H, H_{22}), 4.14 (m, 4H, H_{5+20}), 3.77 – 3.61 (m, 3H, H_{12+16}), 3.48 – 3.38 (m, 4H, H_{20+23}), 2.87 – 2.79 (m, 1H, H_{15}), 2.10 (m, 3H, H_{13+19}), 2.00 – 1.92 (m, 1H, H_{14}), 1.36 (m, 1H, H_{13}). **^{13}C NMR** (151 MHz, DMSO-d_6) δ 159.3 (C_4), 154.9 (C_1), 146.3 (C_{10}), 137.9 (C_{17}), 133.6 (C_8), 127.1 (C_7), 126.6 (C_3), 121.8 (C_2), 121.1 (C_9), 116.6 (C_{18}), 103.9 (C_6), 66.2 (C_{12}), 64.5 (C_{11}), 64.2 (C_{16}), 56.3 (C_5), 54.4 (C_{20}), 48.8 (C_{23}), 45.6 (C_{22}), 37.4 (C_{15}), 25.8 (C_{14}), 24.6 (C_{19}), 19.4 (C_{13}). **^{19}F NMR** (376 MHz, CD_3OD) δ -150.49 (m, ^{10}B), -150.54 (m, ^{11}B). **HR-ESI MS** m/z = 440.2379 $[M-BF_4]^+$ (calculated for $C_{22}H_{30}N_2O_2{}^{10}BF_4$ = 440.2373).

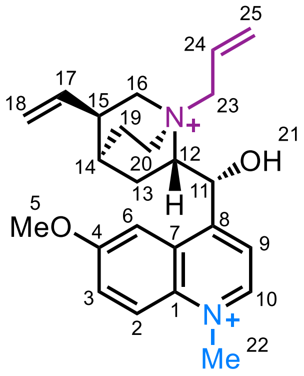

1·IBr: Quinine (500 mg, 1.54 mmol) was placed into an oven-dried round-bottomed flask. CH_2Cl_2 (2.5 mL) was added and stirred at rt before adding allyl bromide (0.13 mL, 1.54 mmol). The resulting solution was stirred at rt for 16 h. Once the reaction was complete, the volatiles were removed under vacuum. The resulting solid was dissolved in MeCN (2.5 mL) and MeI (0.13 mL, 2.09 mmol) was added dropwise and the mixture was heated to 100 °C for 3 h. The mixture was allowed to cool to rt and further cooled to 5 °C in a fridge to allow the product to precipitate. The orange precipitate was isolated by filtration and washed with CH_2Cl_2 (2 × 5 mL), then dried under high vacuum to give the title compound as an orange solid (710 mg, 1.25 mmol, 81%). **M.P**. 203 – 205 °C. **^{1}H NMR** (400 MHz, DMSO-d_6) δ 9.32 (d, J = 6.1 Hz, 1H, H_{10}), 8.50 (d, J = 9.7 Hz, 1H, H_2), 8.25 (d, J = 6.0 Hz, 1H, H_9), 8.00 (dd, J = 9.7, 2.7 Hz, 1H, H_3), 7.54 (d, J = 2.7 Hz, 1H, H_6), 7.10 (d, J = 4.1 Hz, 1H, H_{21}), 6.55 (m, 1H, H_{11}), 6.26 (m, 1H, H_{24}), 5.81 – 5.59 (m, 3H, H_{17+23}), 5.09 –

4.97 (m, 2H, H_{18}), 4.58 (s, 3H, H_{22}), 4.54 (m, 1H, H_{25}) 4.29 (m, 1H, H_{25}), 4.12 (s, 3H, H_5), 4.05 – 3.96 (m, 1H, H_{20}), 3.76 – 3.65 (m, 2H, H_{12+16}), 3.57 – 3.39 (m, 2H, H_{16+20}), 2.79 (m, 1H, H_{15}), 2.13 – 1.96 (m, 4H, $H_{13+14+19}$), 1.32 (m, 1H, H_{13}). **^{13}C NMR** (101 MHz, DMSO-*d₆*) δ 159.4 (C_4), 154.9 (C_1), 146.4 (C_{10}), 138.1 (C_{17}), 133.7 (C_8), 127.5 (C_{23}), 127.3 (C_7), 126.6 (C_3), 126.2 (C_{24}), 121.9 (C_2), 121.5 (C_9), 116.7 (C_{18}), 104.4 (C_6), 65.9 (C_{12}), 64.6 (C_{11}), 62.5 (C_{25}), 60.3 (C_{16}), 56.5 (C_5), 52.2 (C_{20}), 45.7 (C_{22}), 37.3 (C_{15}), 26.1 (C_{14}), 24.5 (C_{19}), 20.4 (C_{13}). **HR-ESI-MS** *m/z* = 507.1505 $[M-Br]^+$ (calculated for $C_{24}H_{32}N_2O_2I$ = 507.1508).

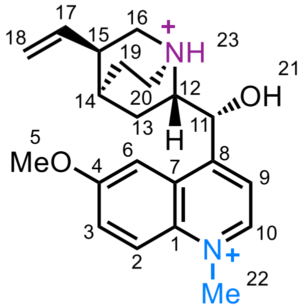

H-iMeQn·2Cl: **1**·IBr (80 mg, 0.14 mmol) was dissolved in Me_2SO (1 mL) then $Pd(PPh_3)_4$ (8 mg, 6.92 µM, 5 mol%) and barbituric acid (52 mg, 0.4 mmol) were added to the reaction mixture and stirred overnight at 40 °C. A 1 M aqueous solution of HCl (1 mL) was added and stirred for 5 min. The mixture was then purified by reverse phase column chromatography (Teledyne Isco CombiFlash Rf+ system, 40 g C18-capped SiO_2, 0.05 M HCl(aq) / MeOH, 0 – 100% elution). After evaporation, the product was isolated as a yellow film (57 mg, 0.14 mmol, quantitative). **^{1}H NMR** (400 MHz, DMSO-*d₆*) δ 9.14 (d, *J* = 6.1 Hz, 1H, H_{10}), 8.45 (d, *J* = 9.8 Hz, 1H, H_2), 8.28 (d, *J* = 6.1 Hz, 1H, H_9), 7.88 (m, 2H, H_{3+6}), 6.51 (s, 1H, H_{11}), 5.75 (ddd, *J* = 17.4, 10.4, 7.2 Hz, 1H, H_{17}), 5.10 – 5.00 (m, 2H, H_{18}), 4.63 (s, 3H, H_{22}), 4.27 – 4.19 (m, 1H, H_{20}), 4.16 (s, 3H, H_5), 3.67 (m, 1H, H_{12}), 3.60 (m, 1H, H_{16}), 3.31 (m, 1H, H_{16}), 3.24 (m, 1H, H_{20}) 2.81 (m, 1H, H_{15}), 2.19 (m, 2H, H_{13+19}), 2.09 (m, 1H, H_{14}), 1.98 (m, 1H, H_{19}), 1.57 (m, 1H, H_{13}). **^{13}C NMR** (101 MHz, DMSO-*d₆*) δ 162.4 (C_4), 158.0 (C_1), 147.1 (C_{10}), 139.2 (C_{17}), 135.9 (C_8), 129.8 (C_7), 129.4 (C_3), 122.4 (C_2), 121.6 (C_9), 117.3 (C_{18}), 104.5 (C_6), 68.1 (C_{11}), 60.9

(C_{12}), 58.6 (C_5), 55.5 (C_{16}), 46.5 (C_{23}), 45.3 (C_{20}), 38.5 (C_{15}), 28.4 (C_{14}), 25.1 (C_{19}), 19.2 (C_{13}). **HR-ESI-MS** *m/z* = 409.1472 [M-H]$^+$ (calculated for $C_{21}H_{27}N_2O_2Cl_2$ = 409.1449).

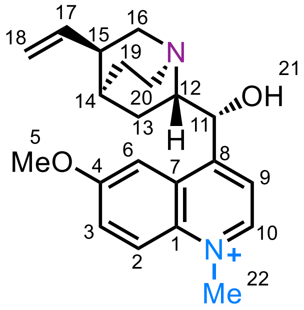

iMeQn·BF_4: **H-iMeQn**·2Cl (64 mg, 0.16 mmol) was dissolved in MeOH (10 mL) and diisopropylaminomethyl polystyrene (467 mg, 1.4 mmol, 200–400 mesh particle size, extent of labelling: ~3 mmol/g base loading, matrix crosslinked with 2% divinylbenzene) was added. The reaction mixture was stirred for 1 h at rt before being filtered. The filtrate was evaporated to dryness and washed with hexanes (5 × 10 mL) to extract any solubilised polymer. The residual solid was dried under high vacuum to yield **iMeQn**·Cl (45 mg, 0.12 mmol, 86%). The chloride salt was dissolved in MeOH (3 mL) and a solution of $AgBF_4$ (23 mg, 0.12 mmol) in MeOH (2 mL) was added dropwise then stirred for 10 min at rt. The reaction mixture was filtered and the filtrate was evaporated to dryness to yield the title compound as an off white solid (51 mg, 0.12 mmol, quantitative). **M.P.** 204 – 206 °C. **^{1}H NMR** (400 MHz, CD_3OD) δ 9.14 (d, *J* = 6.1 Hz, 1H, H_{10}), 8.48 (d, *J* = 9.7 Hz, 1H, H_2), 8.27 (d, *J* = 6.1 Hz, 1H, H_9), 7.92 (d, *J* = 9.7, 2.4 Hz, 1H, H_3), 7.87 (m, 1H, H_6), 6.24 (s, 1H, H_{11}), 5.79 (ddd, *J* = 17.4, 10.4, 7.2 Hz, 1H, H_{17}), 5.10 – 5.01 (m, 2H, H_{18}), 4.65 (s, 3H, H_{22}), 4.16 (s, 3H, H_5), 4.06 (m, 1H, H_{20}), 3.52 (m, 1H, H_{12}), 3.48 (m, 1H, H_{16}), 3.13 (m, 2H, H_{16+20}), 2.70 (m, 1H, H_{15}), 2.14 (m, 3H, H_{13+19}), 2.04 (m, 1H, H_{14}) 1.87 (m, 1H, H_{19}), 1.61 (m, 1H, H_{13}). **^{13}C NMR** (101 MHz, CD_3OD) δ 162.1 (C_4), 147.2 (C_{10}), 140.1 (C_{17}), 135.9 (C_8), 129.9 (C_7), 129.0 (C_3), 122.4 (C_2), 121.5 (C_9), 116.7 (C_{18}), 104.5 (C_6), 69.4 (C_{11}), 61.2 (C_{12}), 57.7 (C_5), 56.2 (C_{16}), 46.4 (C_{22}), 45.2 (C_{20}), 39.2 (C_{15}), 28.5 (C_{14}), 25.9 (C_{19}), 20.2 (C_{13}). **^{19}F NMR** (376 MHz, CD_3OD) δ -153.71 (m, ^{10}B), -153.76 (m, ^{11}B). **HR-ESI-MS** *m/z* = 339.2087 [M-BF_4]$^+$ (calculated for $C_{21}H_{27}N_2O_2$ = 339.2037).

3.1 ^{1}H, ^{13}C and ^{19}F NMR Spectroscopic Characterisation of Synthesised Compounds

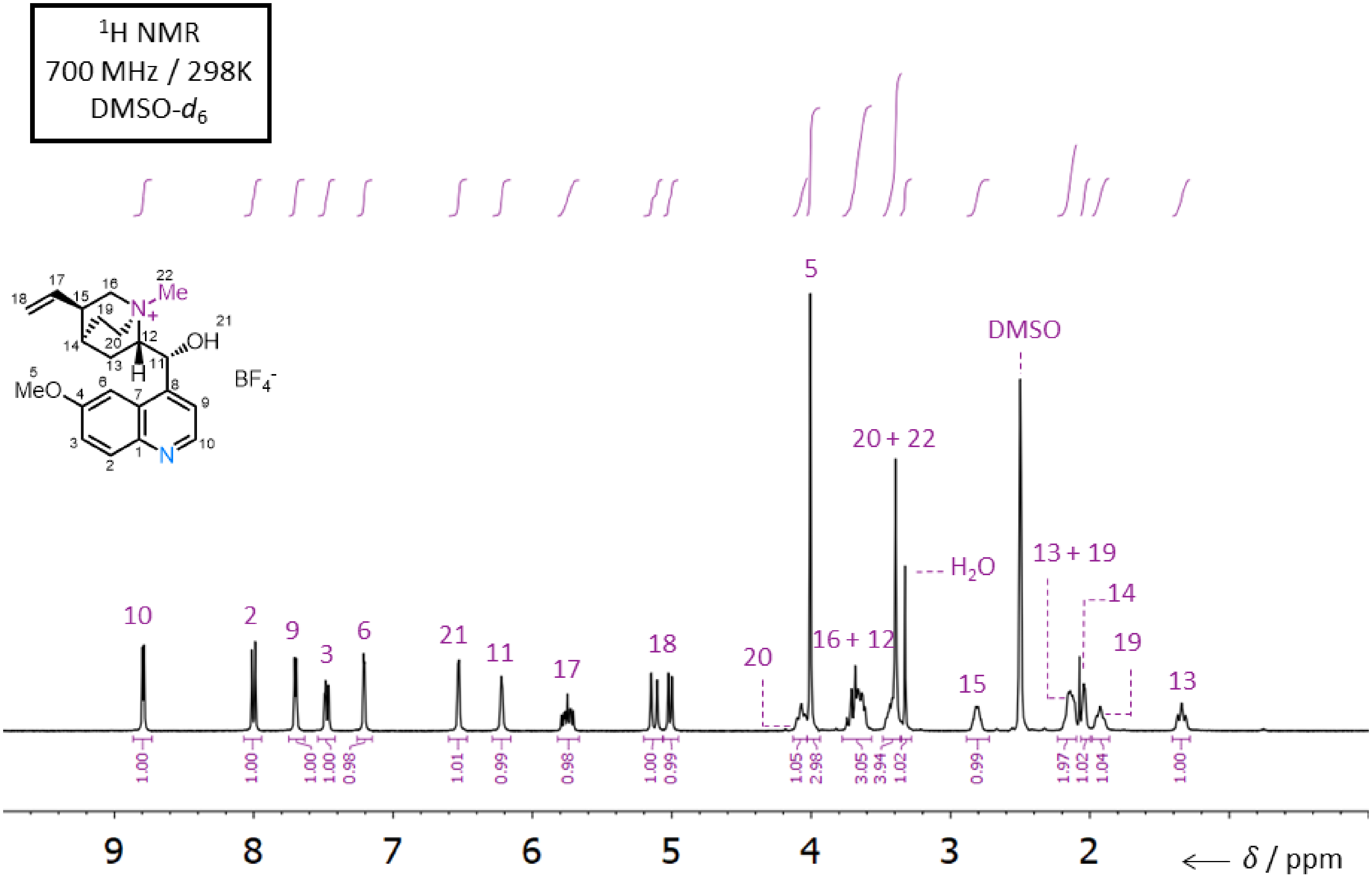

Fig. S1 ^{1}H NMR Spectrum of **MeQn**·BF_4.

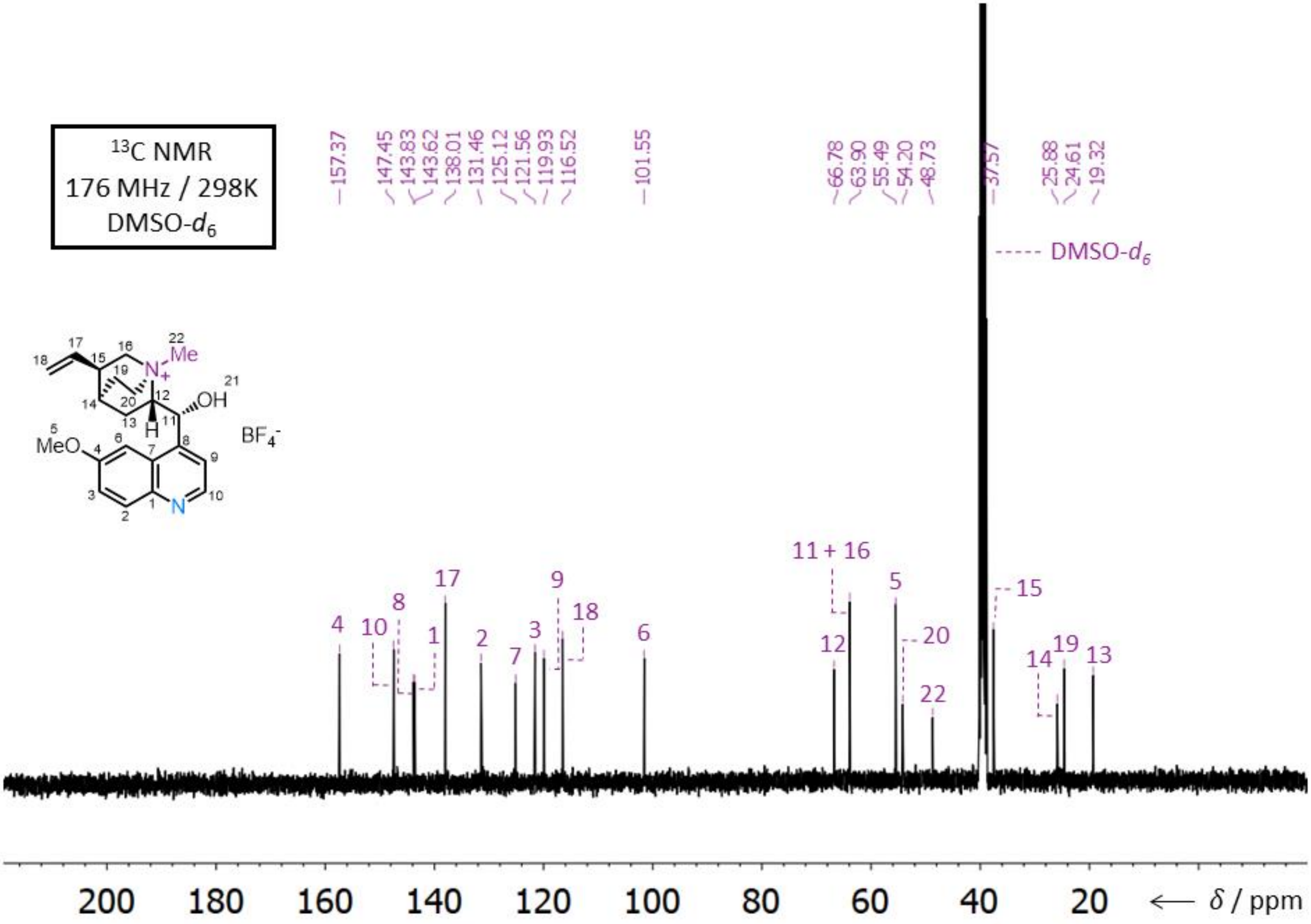

Fig. S2 ^{13}C NMR Spectrum of **MeQn**·BF_4.

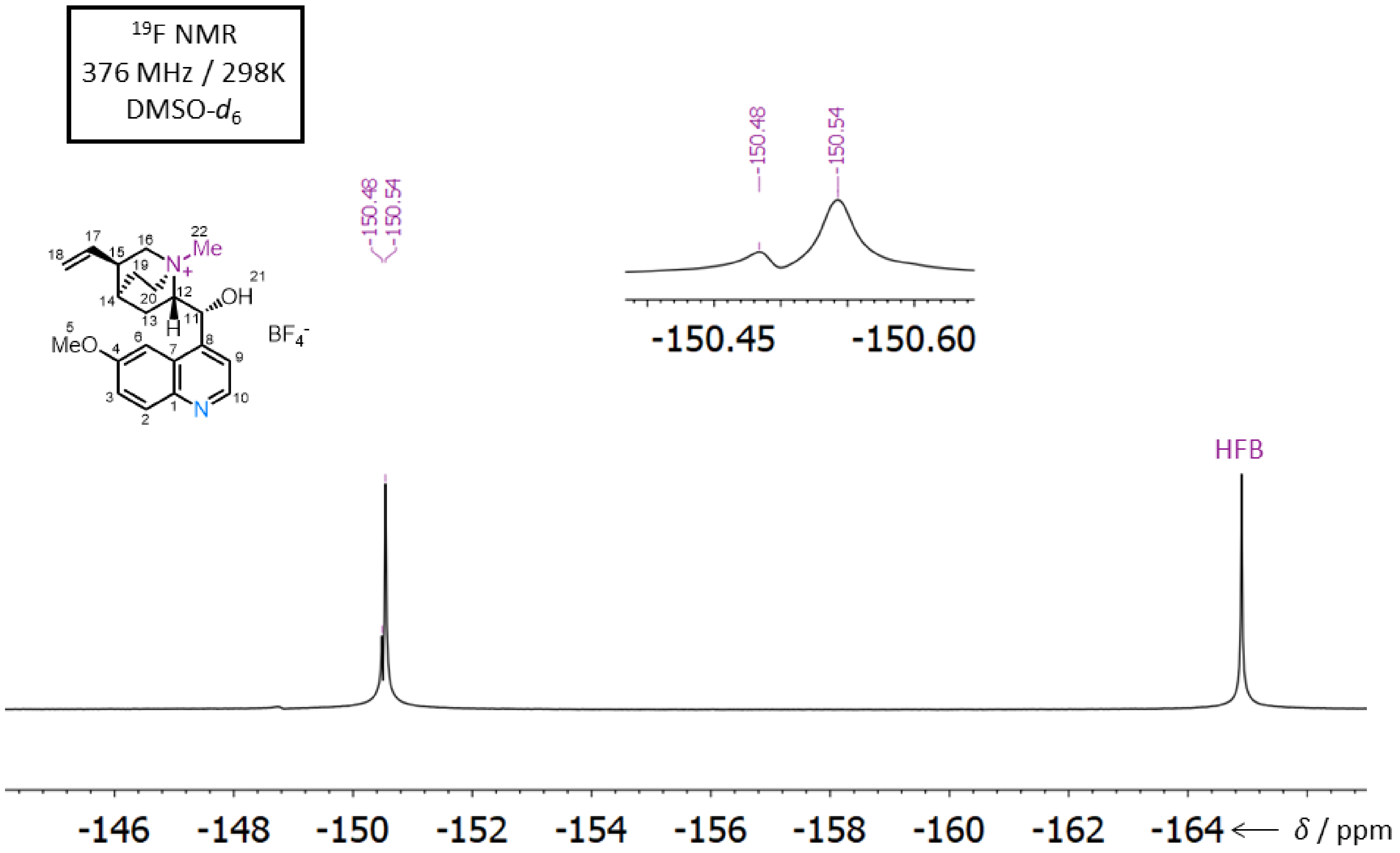

Fig. S3 ^{19}F NMR Spectrum of **MeQn**·BF$_4$.

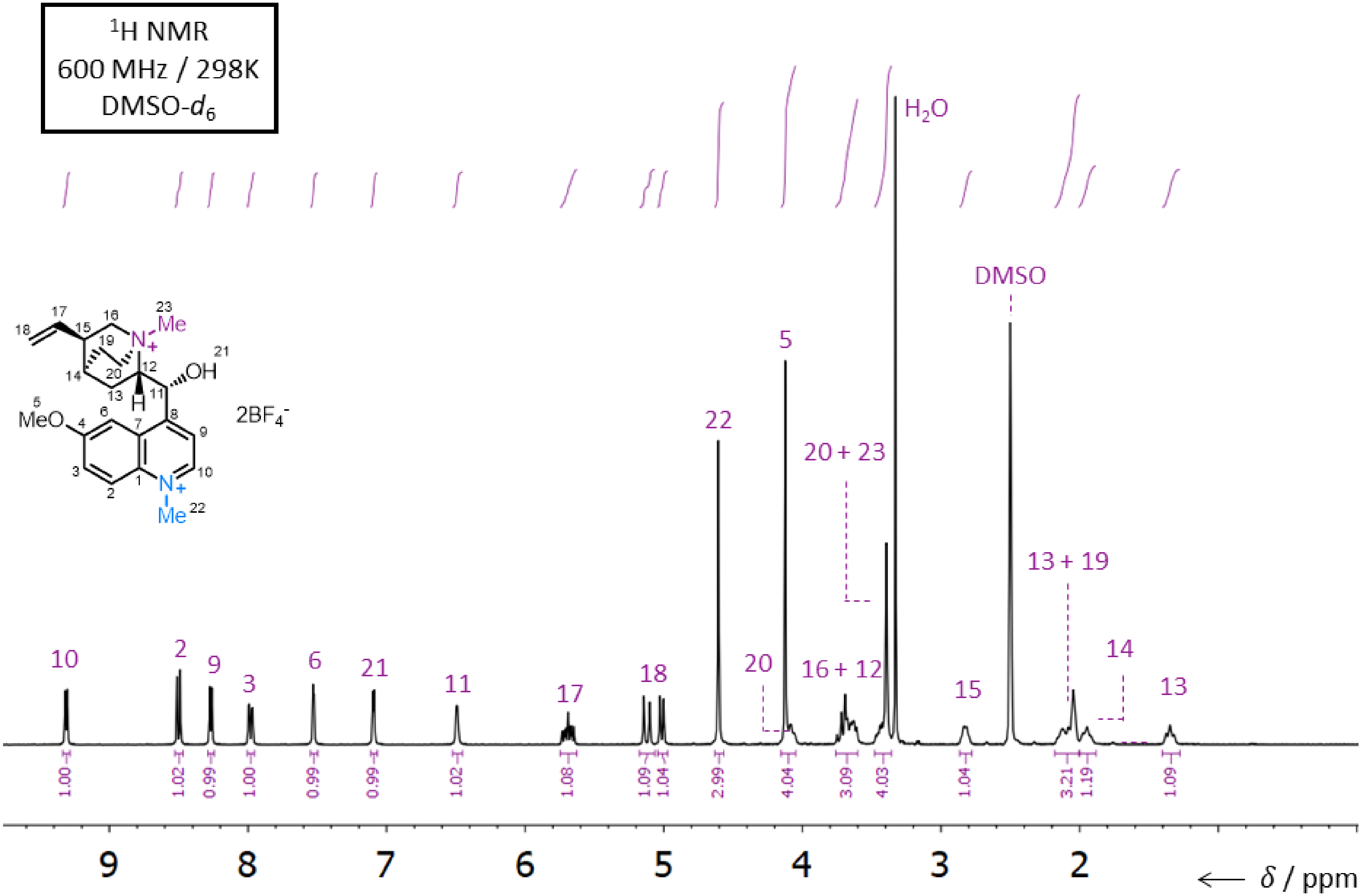

Fig. S4 ^{1}H NMR Spectrum of **Me_2Qn**·BF_4.

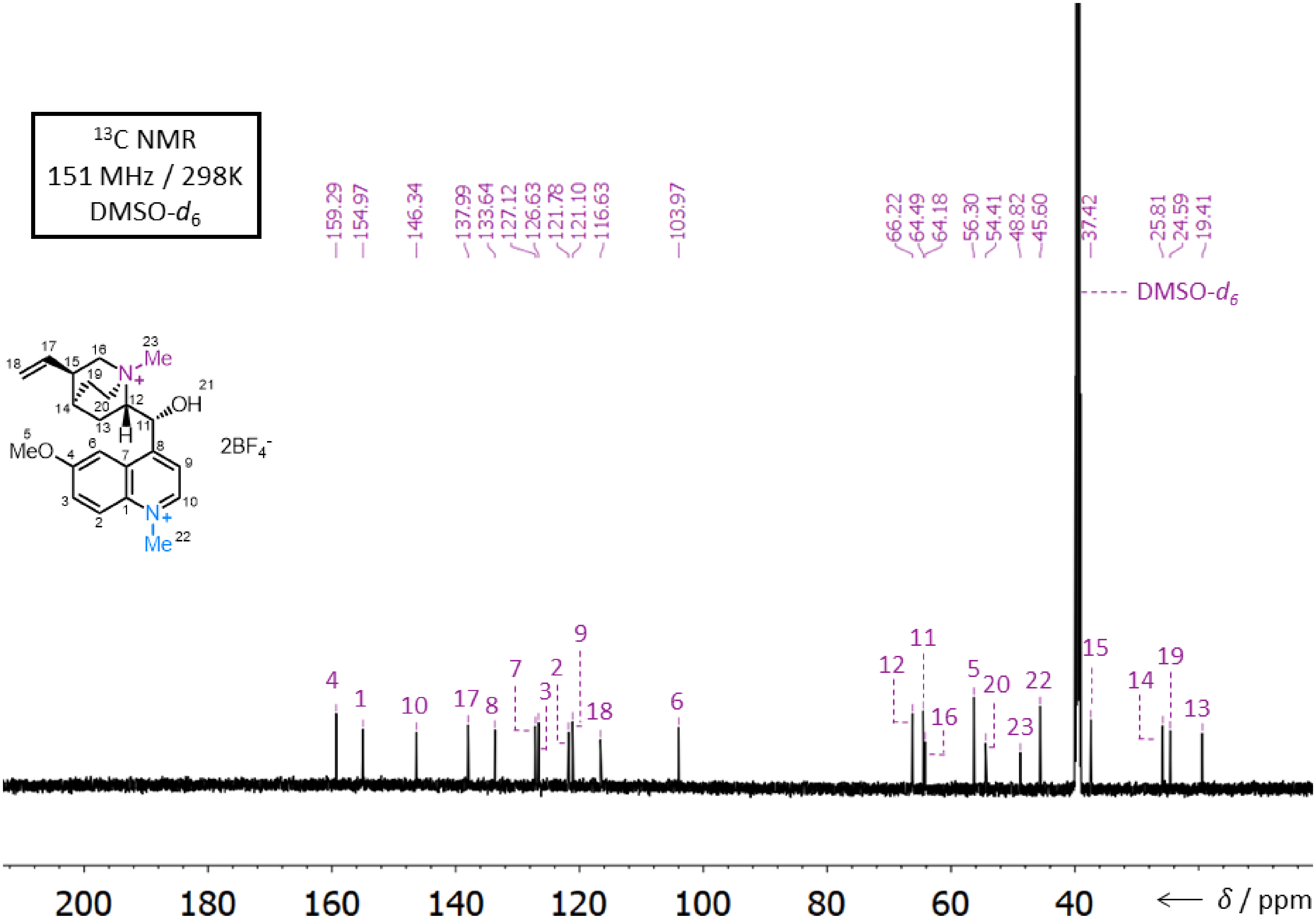

Fig. S5 ^{13}C NMR Spectrum of **Me_2Qn**·BF_4.

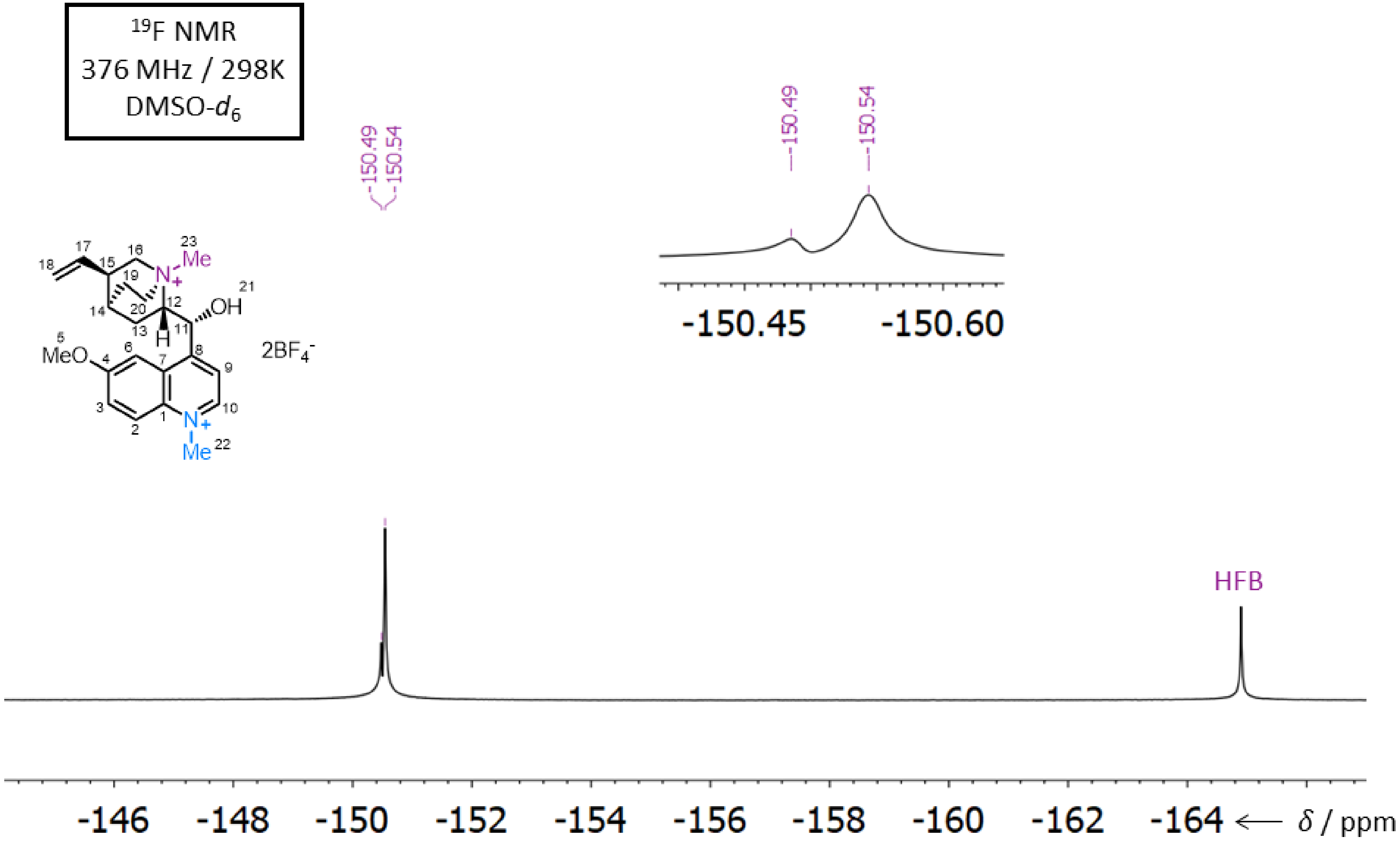

Fig. S6 ^{19}F NMR Spectrum of **Me$_2$Qn**·BF_4.

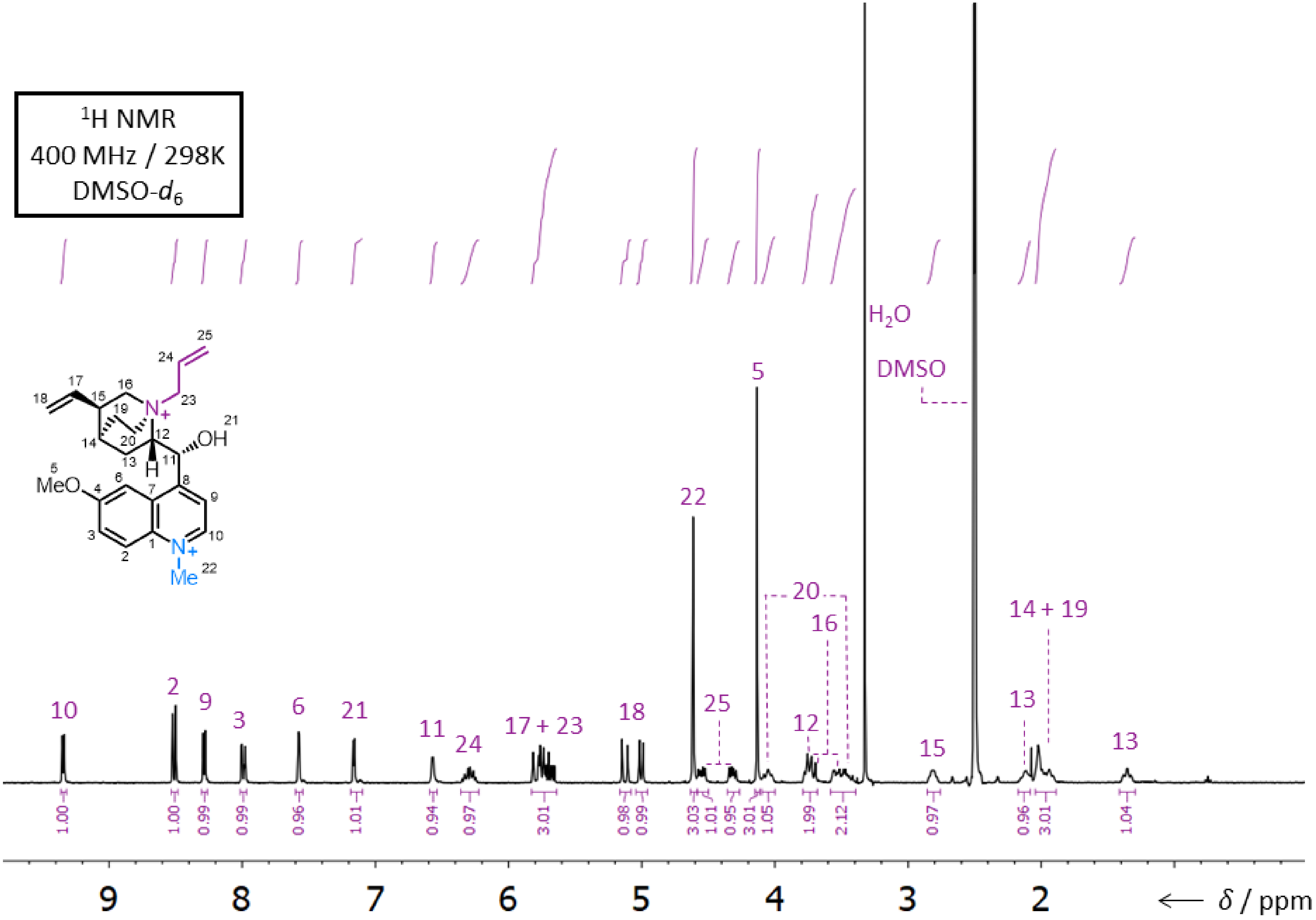

Fig. S7 ^{1}H NMR Spectrum of **1**·2Cl.

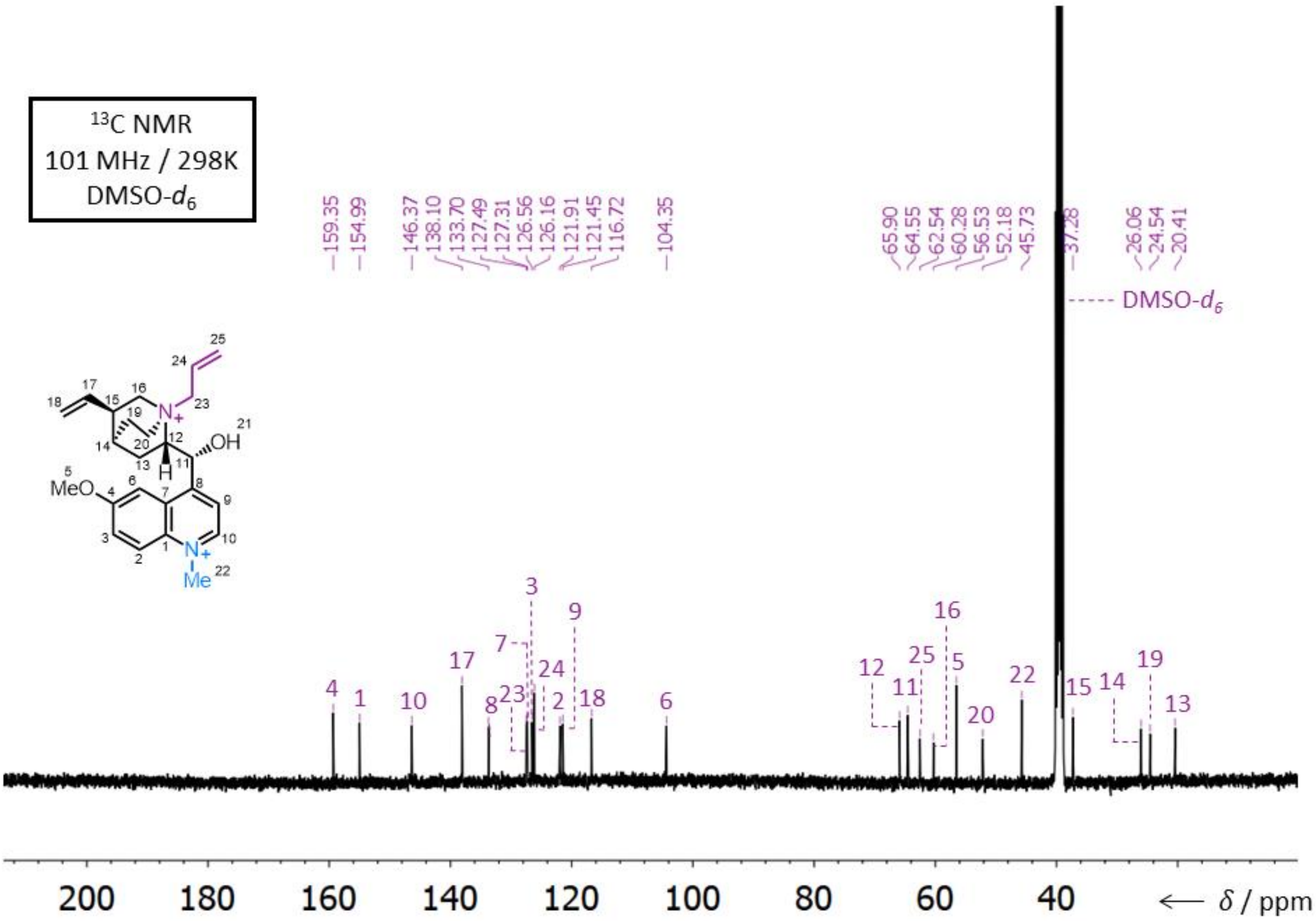

Fig. S8 ^{13}C NMR Spectrum of **1**·2Cl.

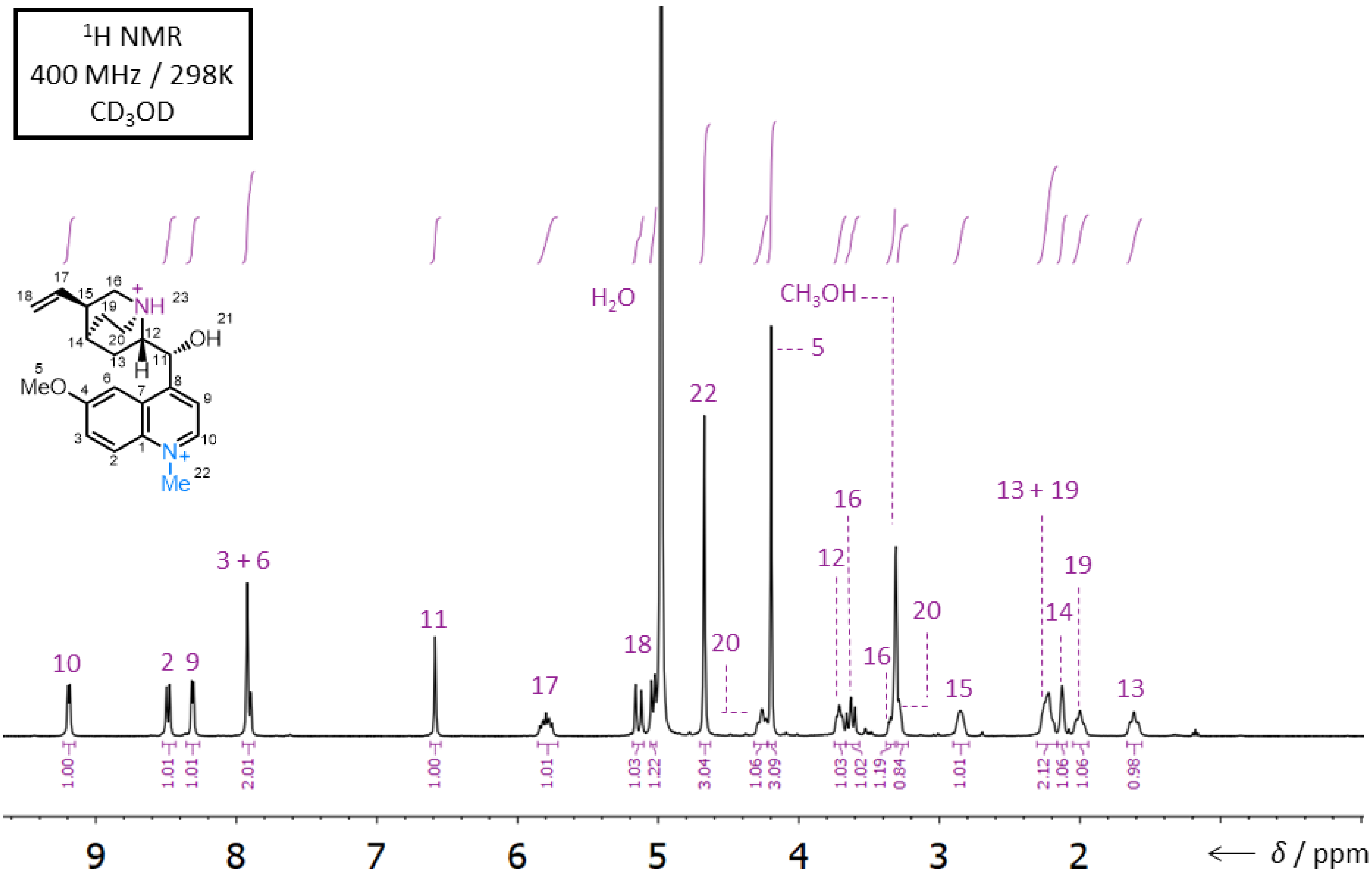

Fig. S9 ^{1}H NMR Spectrum of **H-iMeQn**·2Cl.

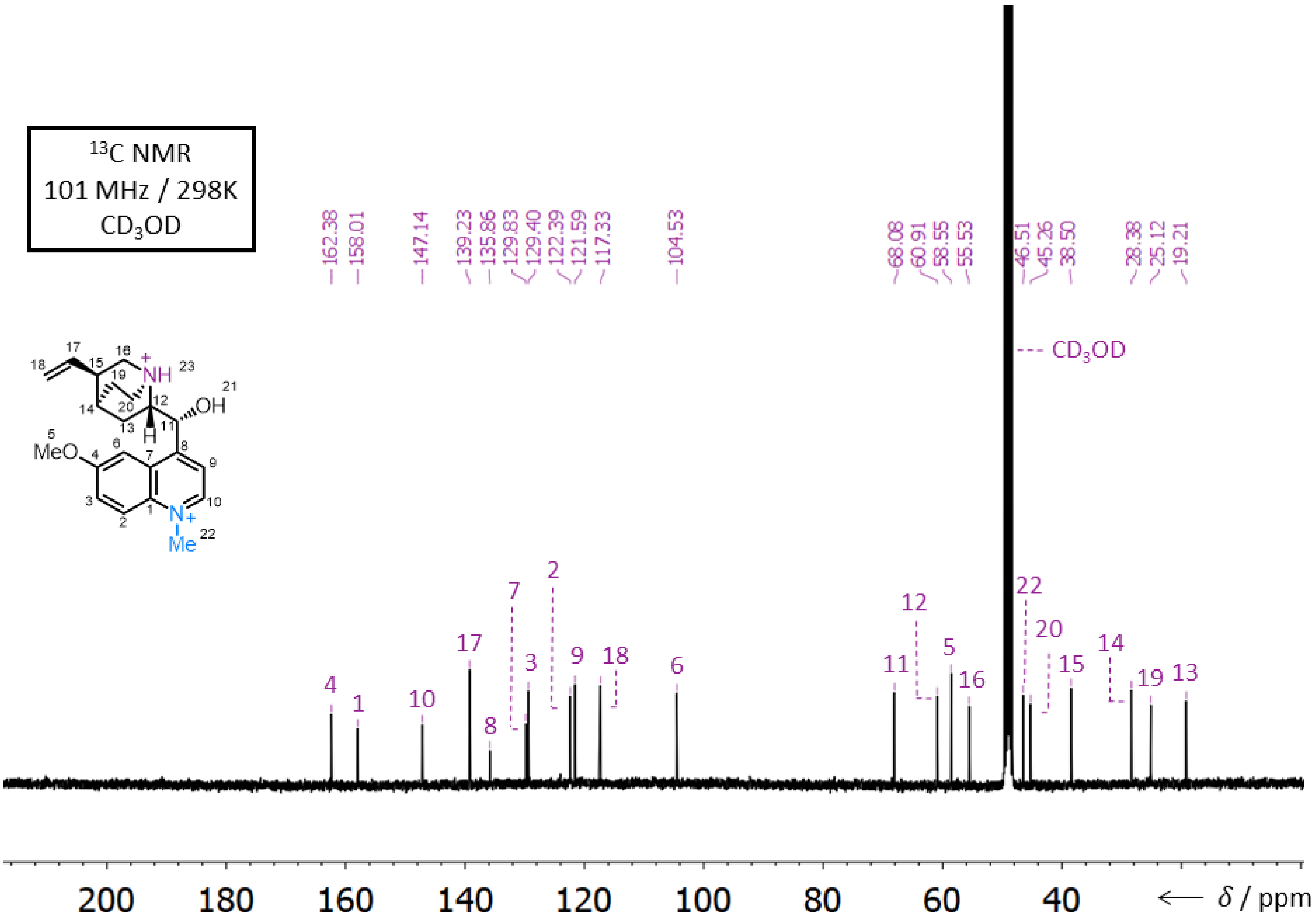

Fig. S10 ^{13}C NMR Spectrum of **H-iMeQn**·2Cl.

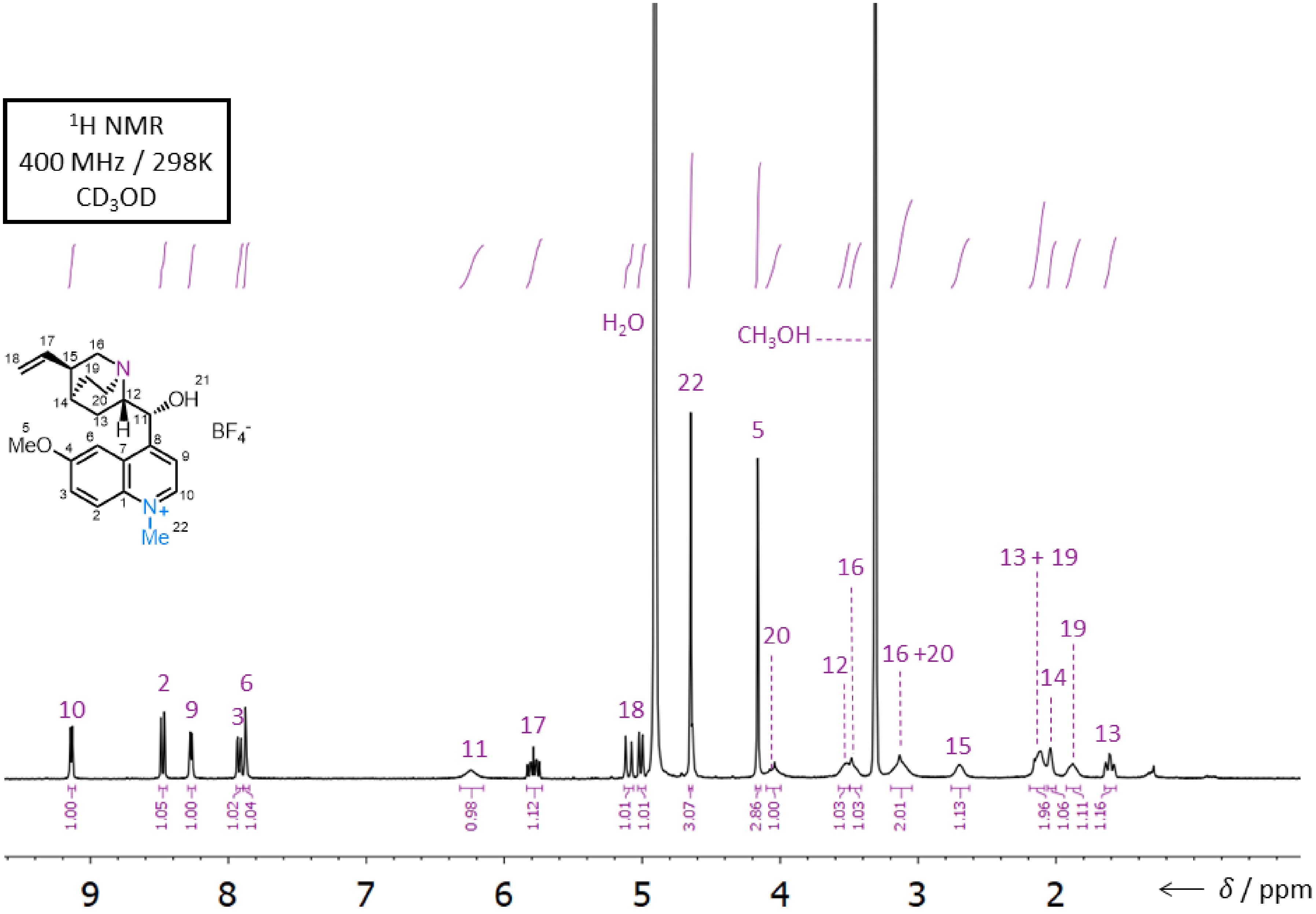

Fig. S11 ^{1}H NMR Spectrum of **iMeQn**·BF_4.

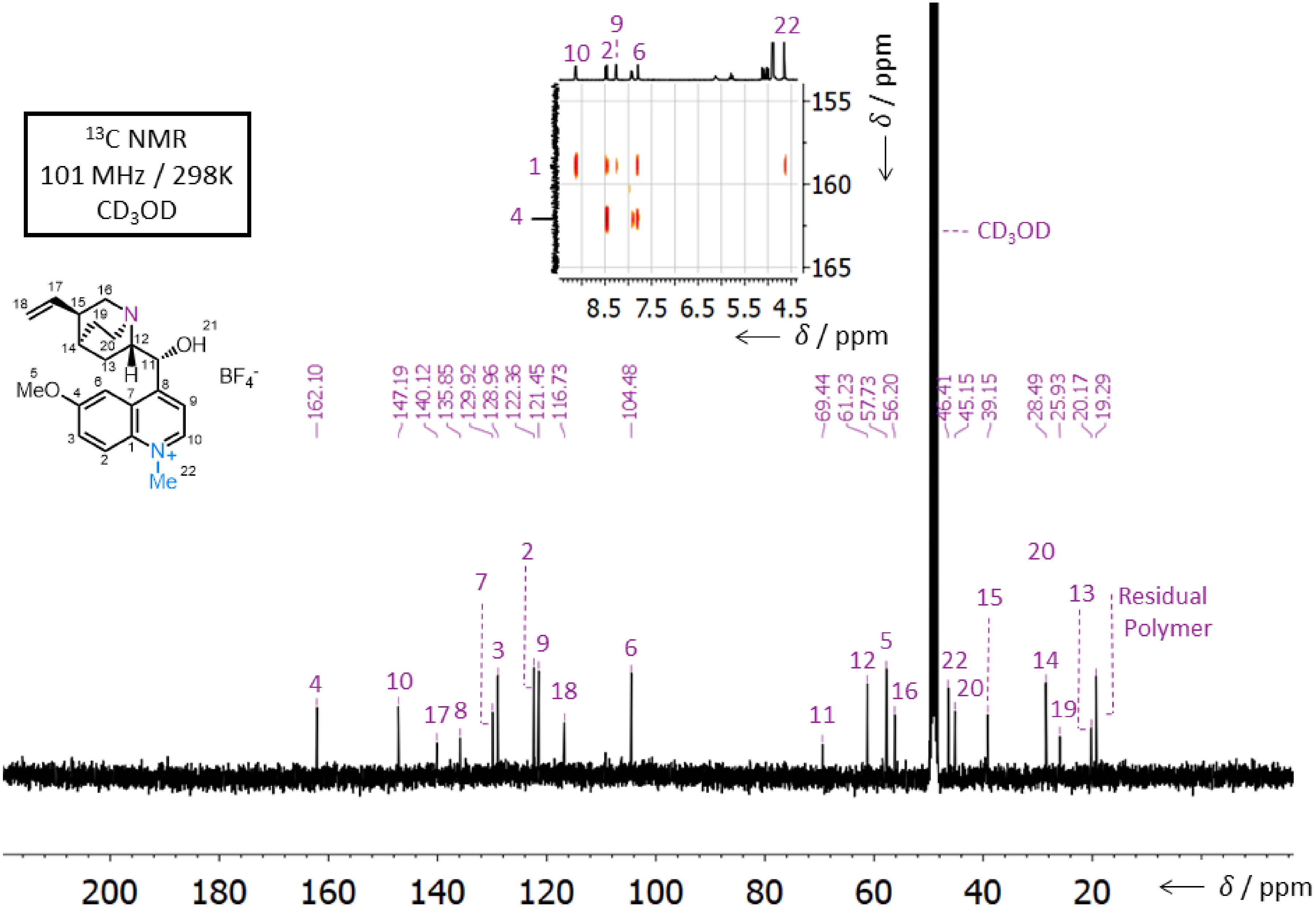

Fig. S12 ^{13}C NMR Spectrum of **iMeQn**·BF_4. Insert: Partial HMBC spectrum to show the ^{13}C resonance for C_1, which is not visible in the 1D spectrum.

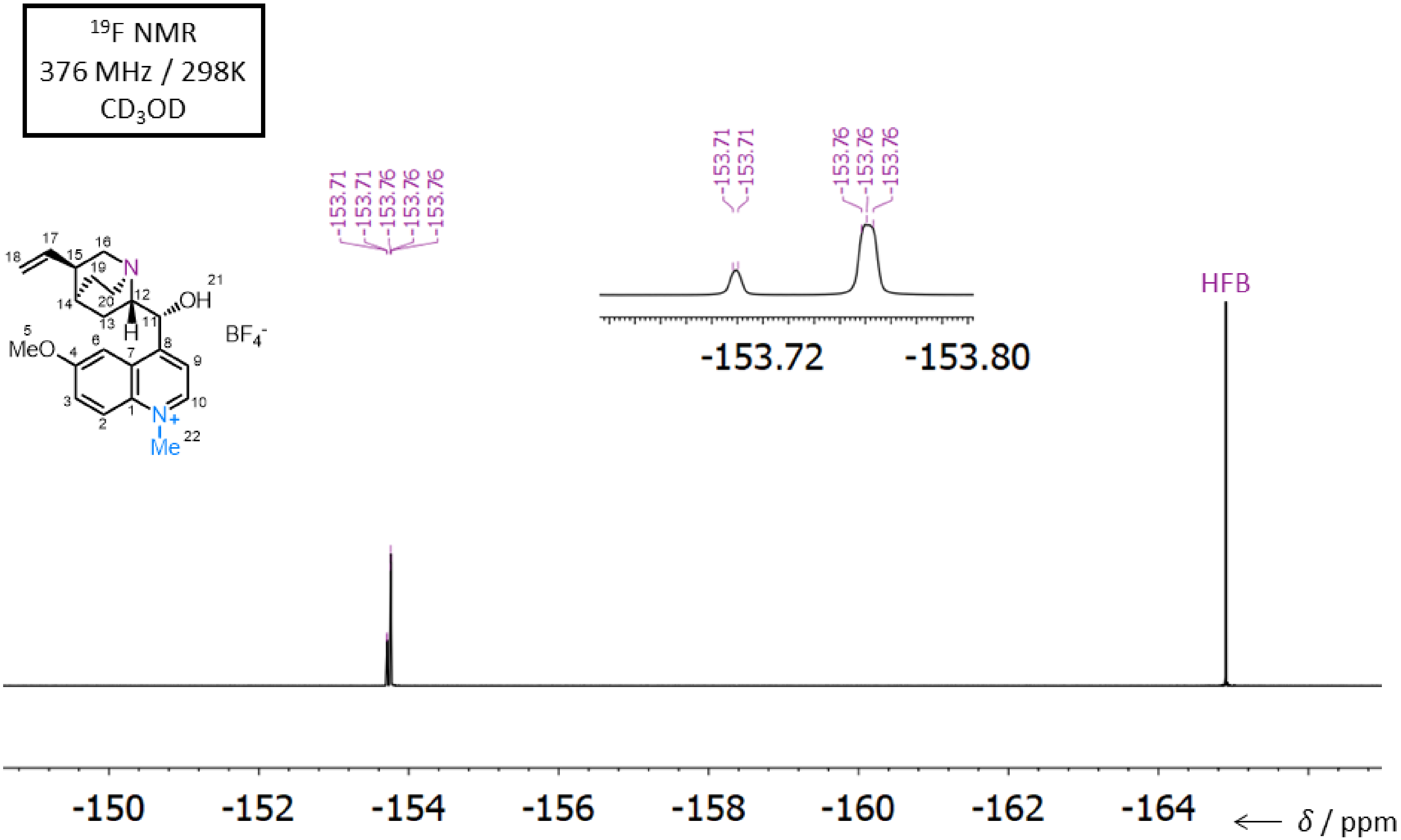

Fig. S13 ^{19}F NMR Spectrum of **iMeQn**·BF_4.

3.2. NMR Comparisons of Methylated and Protonated Quinines

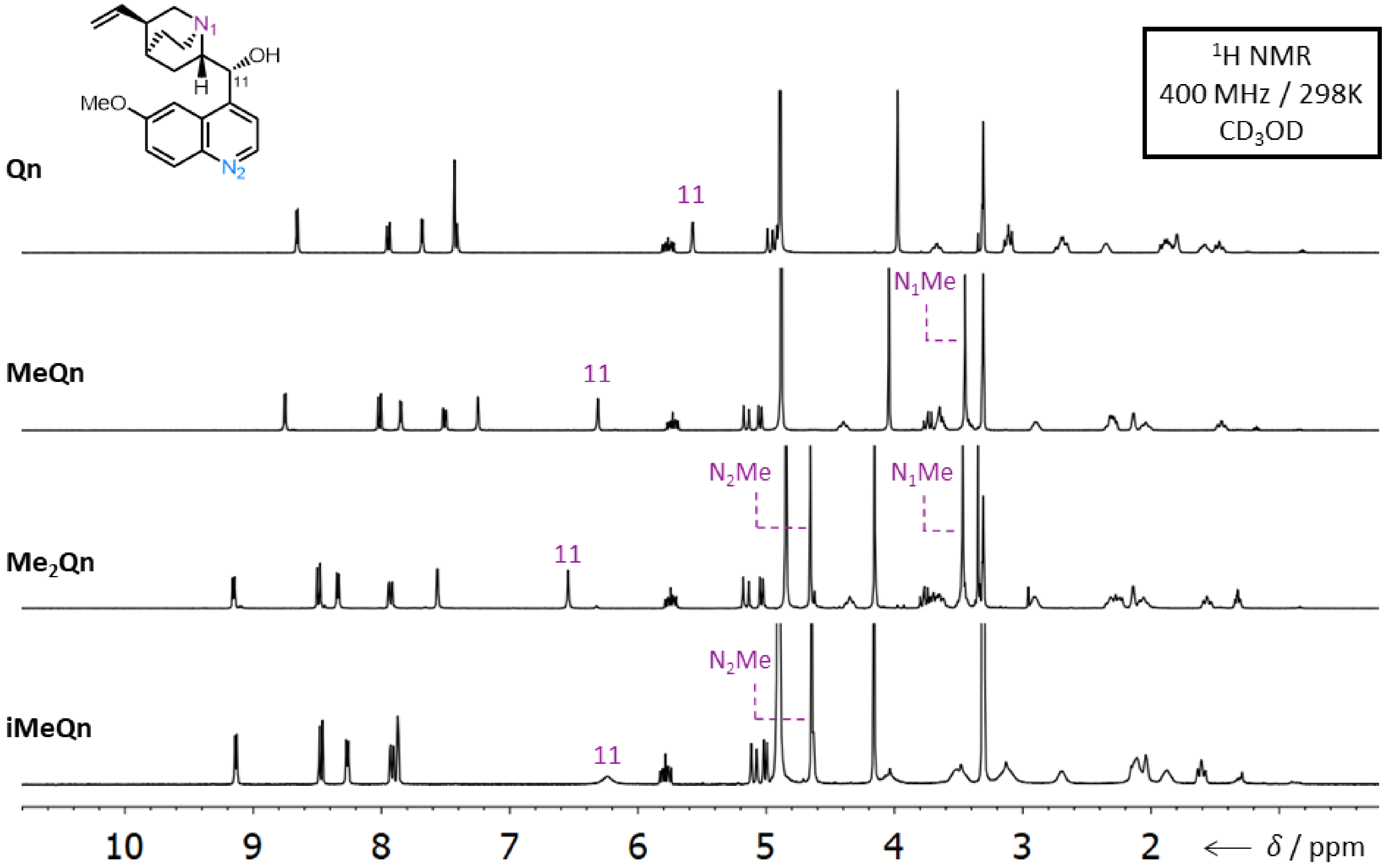

Fig. S14 Comparison of ^{1}H NMR spectra of **Qn**, **MeQn**·BF_4, **Me$_2$Qn**·2BF_4 and **iMeQn**·BF_4, demonstrating the deshielding of H_{11} upon alkylation and the characteristic Me resonances observed for the two N sites.

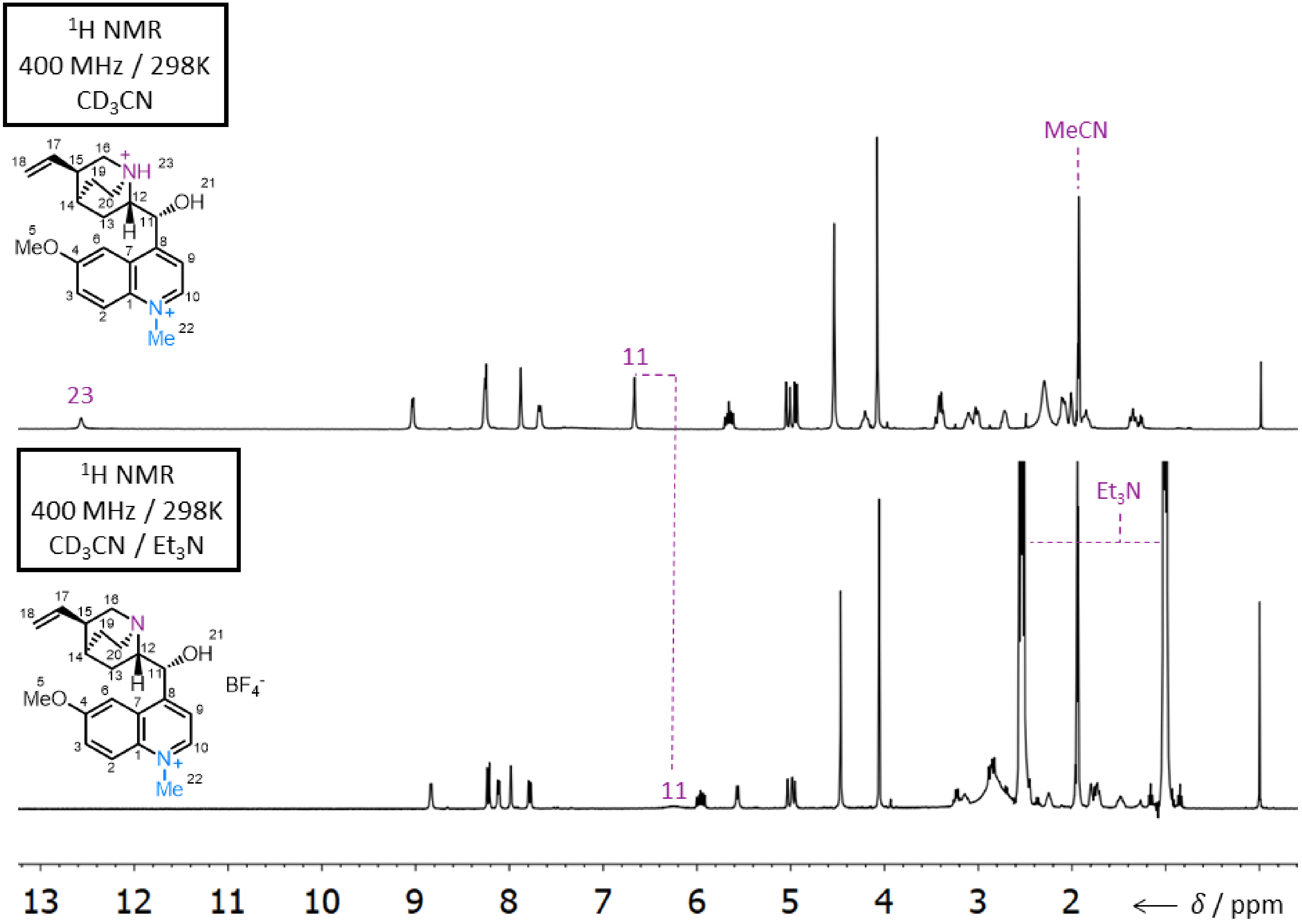

Fig. S15 Comparison of **H-iMeQn**·$2BF_4$ and **iMeQn**·BF_4 ^{1}H NMR spectra. The shift in the resonance of H_{11} is consistent with spectroscopic data in the literature for (de)protonation at N_1[8] and with the shifts observed (Figure S14) upon methylation.

4. X-Ray Crystallographic Analysis

4.1 **MeQn**·BF_4

Crystals of **MeQn**·BF_4 suitable for X-ray diffraction were grown by slow cooling of a saturated MeCN solution.

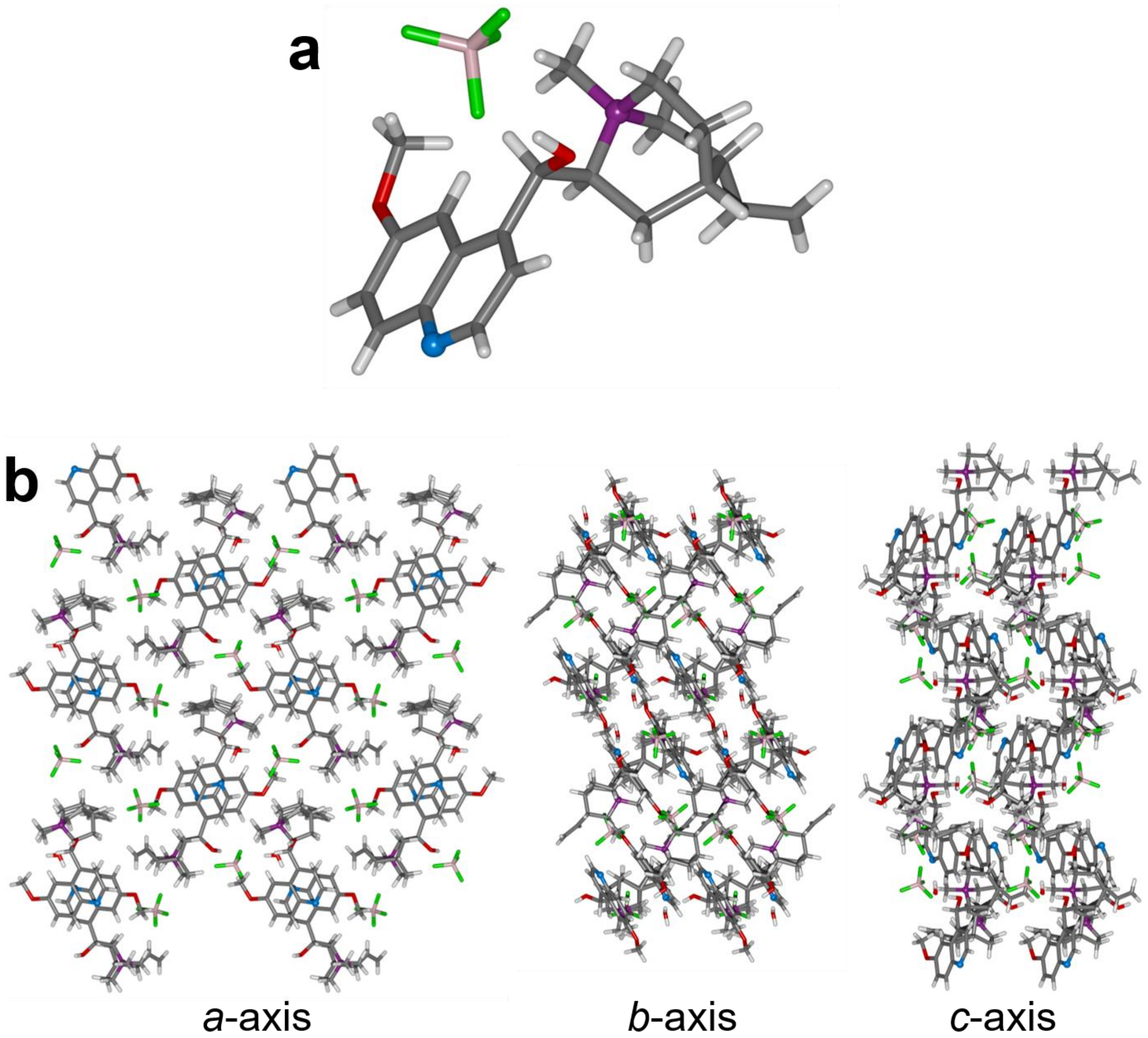

Fig. S16 (a) Solid-state structure and (b) crystal packing of **MeQn**·BF_4.

4.2 $Me_2Qn\cdot 2BF_4$

Crystals of **Me_2Qn**$\cdot 2BF_4$ suitable for X-ray diffraction were grown by slow cooling of a saturated MeCN solution.

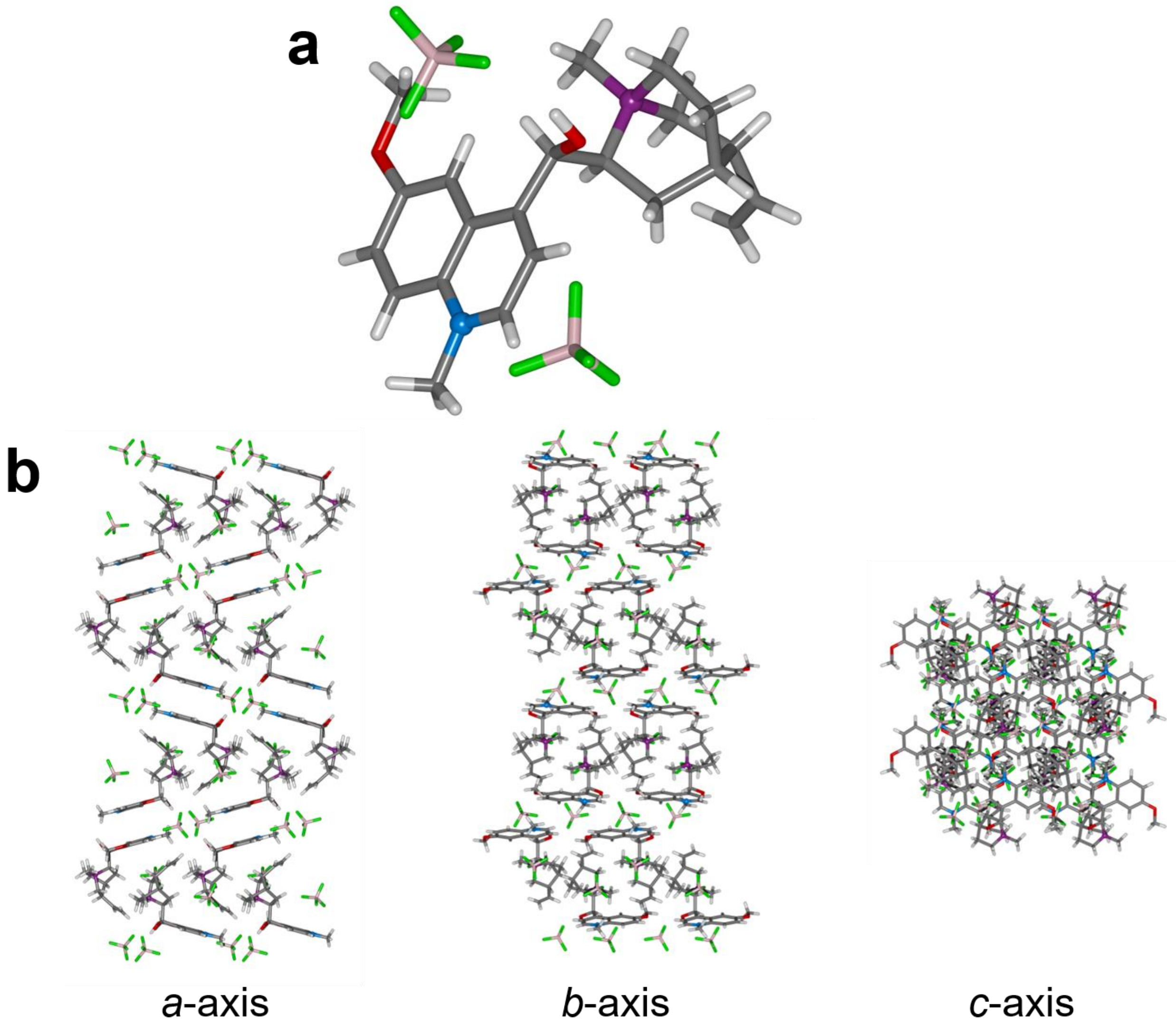

Fig. S17 (a) Solid-state structure and (b) crystal packing of **Me_2Qn**$\cdot 2BF_4$.

Table S1 Crystal Data and Refinement.

Compound	**MeQn**·BF_4	**Me$_2$Qn**·2BF_4
Empirical formula	$C_{21}H_{27}BF_4N_2O_2$ x 0.5 H_2O	$C_{22}H_{30}B_2F_8N_2O_2$
Formula weight	435.26	528.10
Temperature/K	120.0	120.0
Crystal system	monoclinic	orthorhombic
Space group	$P2_1$	$P2_12_12_1$
a/Å	8.0721(2)	9.6961(3)
b/Å	15.6353(5)	10.2407(3)
c/Å	16.4422(5)	23.6533(8)
α/°	90	90
β/°	98.2832(14)	90
γ/°	90	90
Volume/Å^3	2053.52(10)	2348.65(13)
Z	4	4
ρ_{calc} g/cm^3	1.408	1.494
μ/mm^{-1}	0.983	1.195
F(000)	916.0	1096.0
Crystal size/mm^3	0.42 × 0.26 × 0.09	0.39 × 0.28 × 0.12
2Θ range for data collection/°	5.432 to 139.976	7.474 to 144.936
Reflections collected	23769	27372
Independent refl., R_{int}	6963, 0.0308	4554, 0.0314
Data/restraints/parameters	6963/15/575	4554/0/445
Goodness-of-fit on F^2	1.068	1.067
Final R_1 indexes [I≥2σ (I)]	0.0424	0.0256
Final wR_2 indexes [all data]	0.1050	0.0651
Largest diff. peak/hole / e Å^{-3}	0.65/-0.59	0.31/-0.18
Flack parameter	0.01(6)	0.00(3)
Hooft parameter	0.05(4)	-0.01(3)

5. Photoluminescence Quantum Yield Measurements

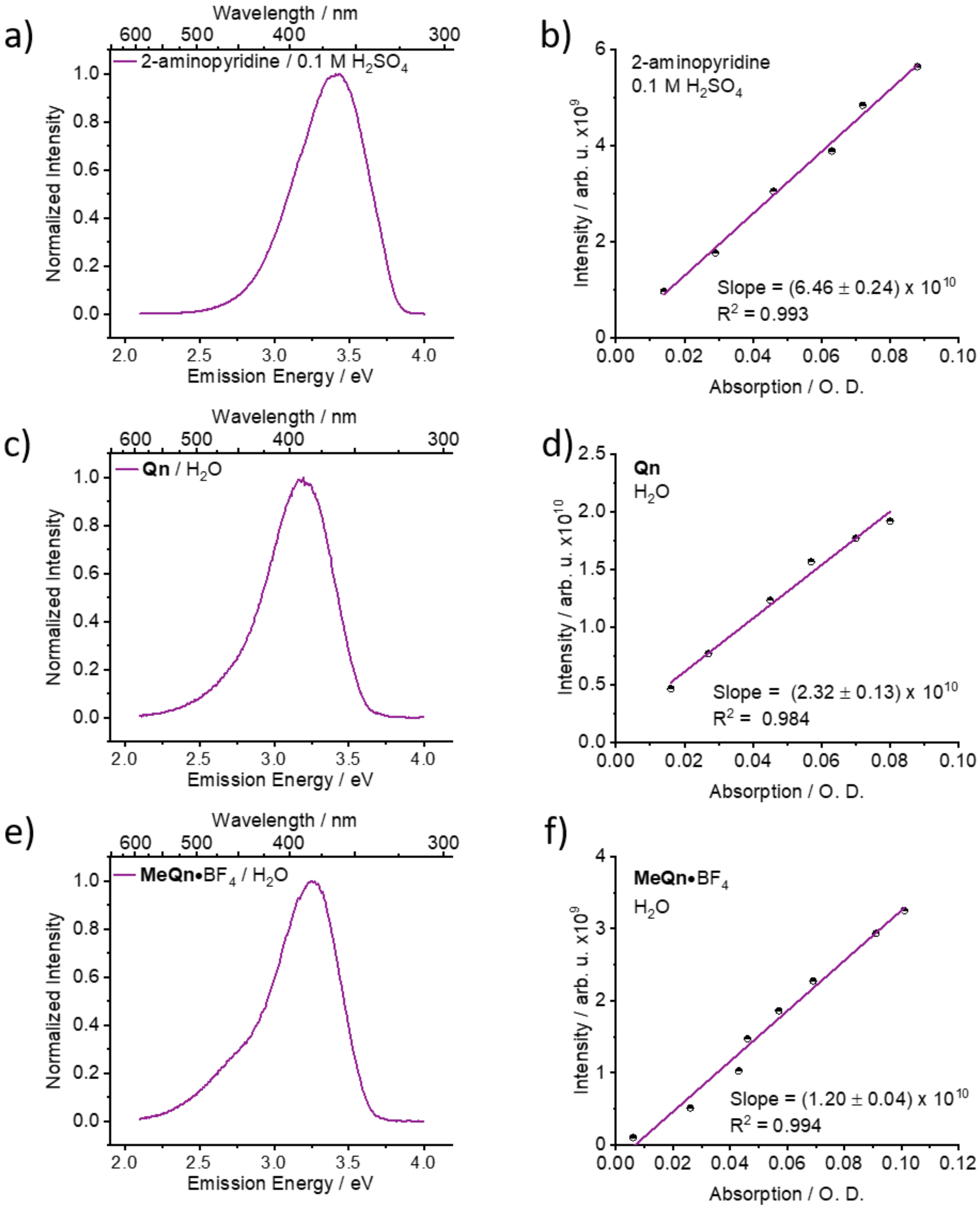

Fig. S18 The emission spectra and emission vs absorption slope of (a-b) 2-aminopyridine in 0.1 M $H_2SO_{4(aq)}$ as a standard, (c-d) **Qn** in H_2O and (e-f) **MeQn**·BF_4 in H_2O.

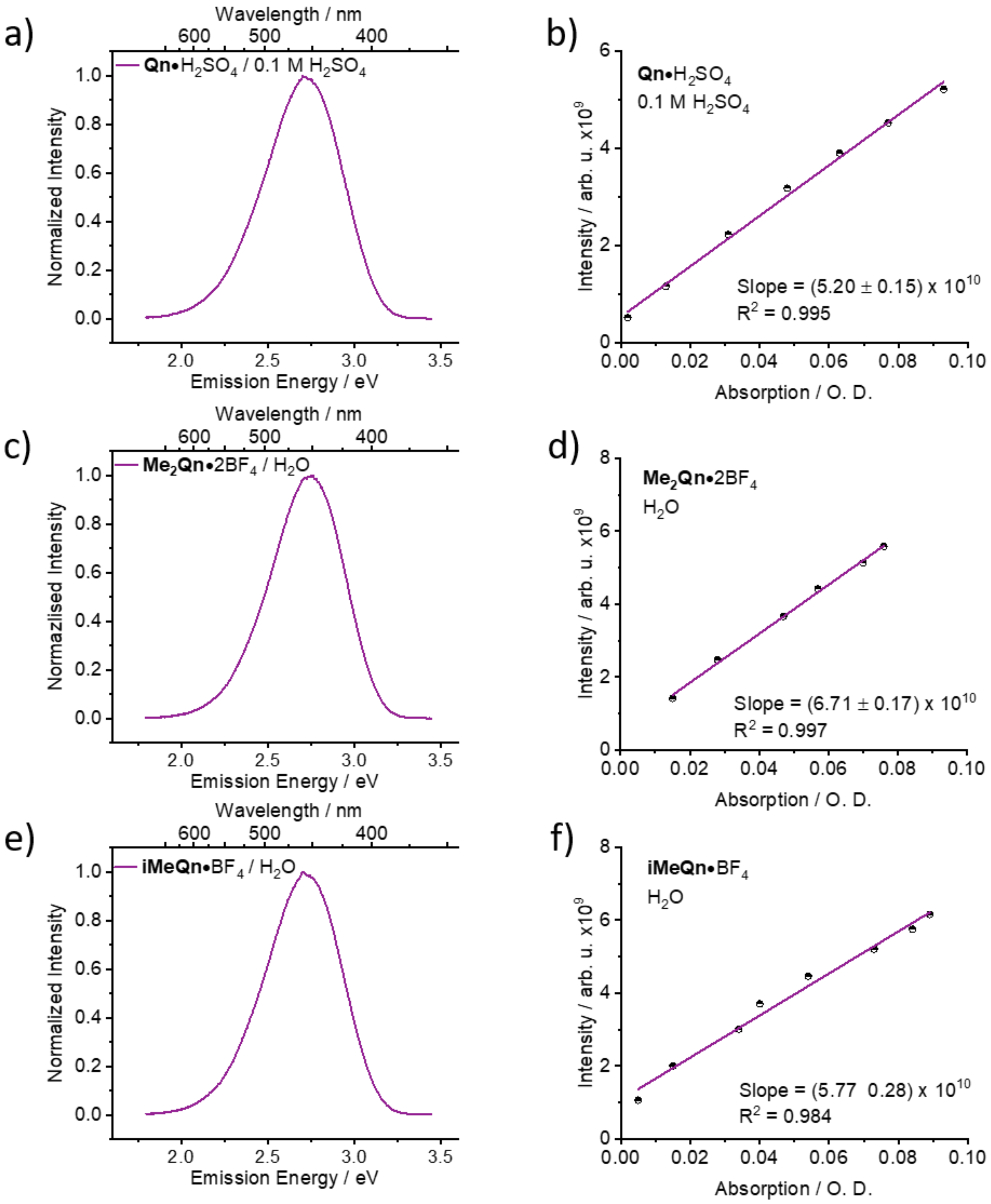

Fig. S19 The emission spectra and emission vs absorption slope of (a-b) **Qn**·H_2SO_4 in 0.1 M $H_2SO_{4(aq)}$ as a standard, (c-d) **Me$_2$Qn**·$2BF_4$ in H_2O and (e-f) **iMeQn**·BF_4 in H_2O.

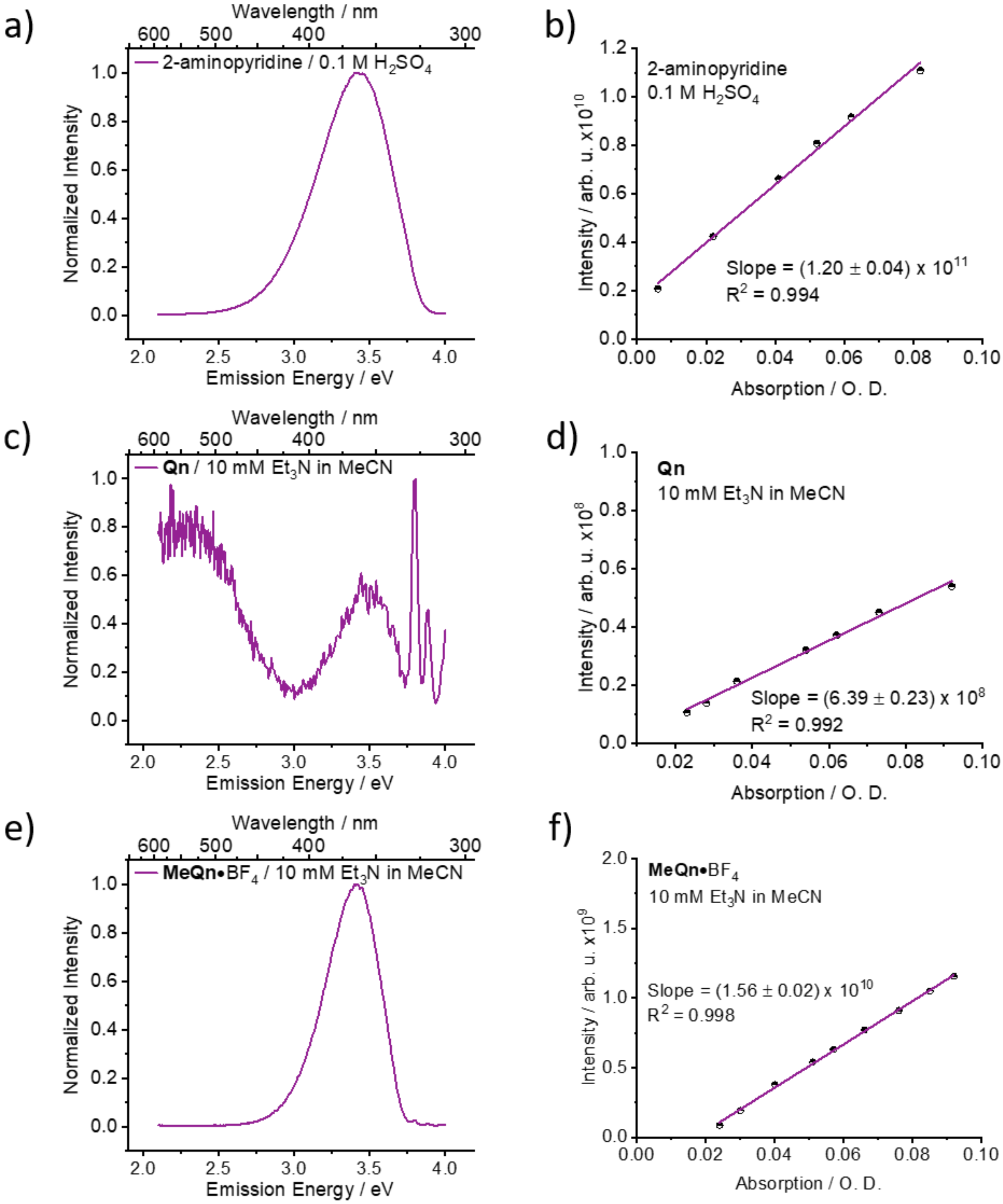

Fig. S20 The emission spectra and emission vs absorption slope of (a-b) 2-aminopyridine in 0.1 M $H_2SO_{4(aq)}$ as a standard, (c-d) **Qn** with 10 mM Et_3N in MeCN and (e-f) **MeQn**·BF_4 with 10 mM Et_3N in MeCN.

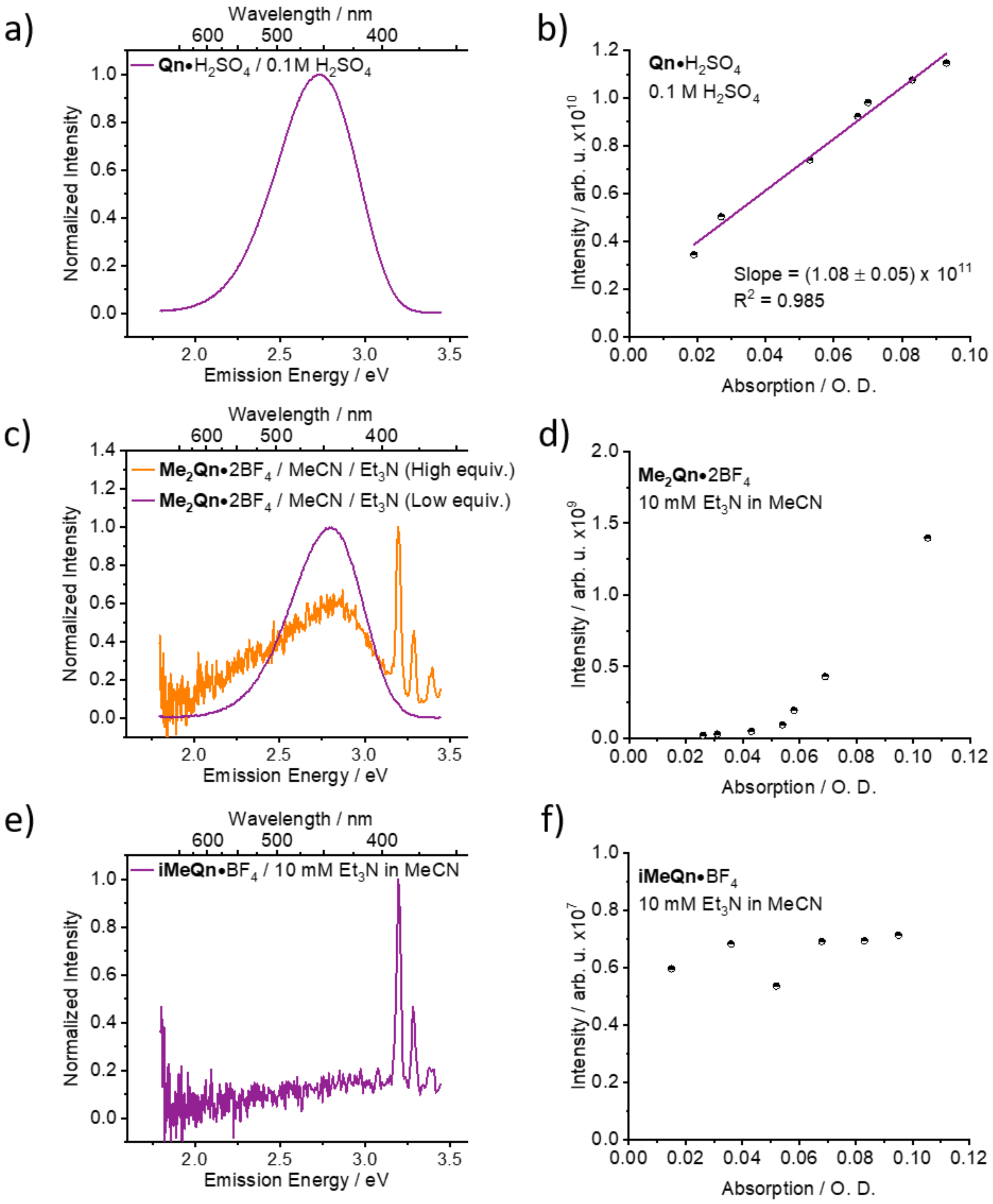

Fig. S21 The emission spectra and emission vs absorption slope of (a-b) **Qn**·H_2SO_4 in 0.1 M $H_2SO_{4(aq)}$ as a standard, (c-d) **Me_2Qn**·$2BF_4$ with 10 mM Et_3N in MeCN and (e-f) **iMeQn**·BF_4 with 10 mM Et_3N in MeCN.

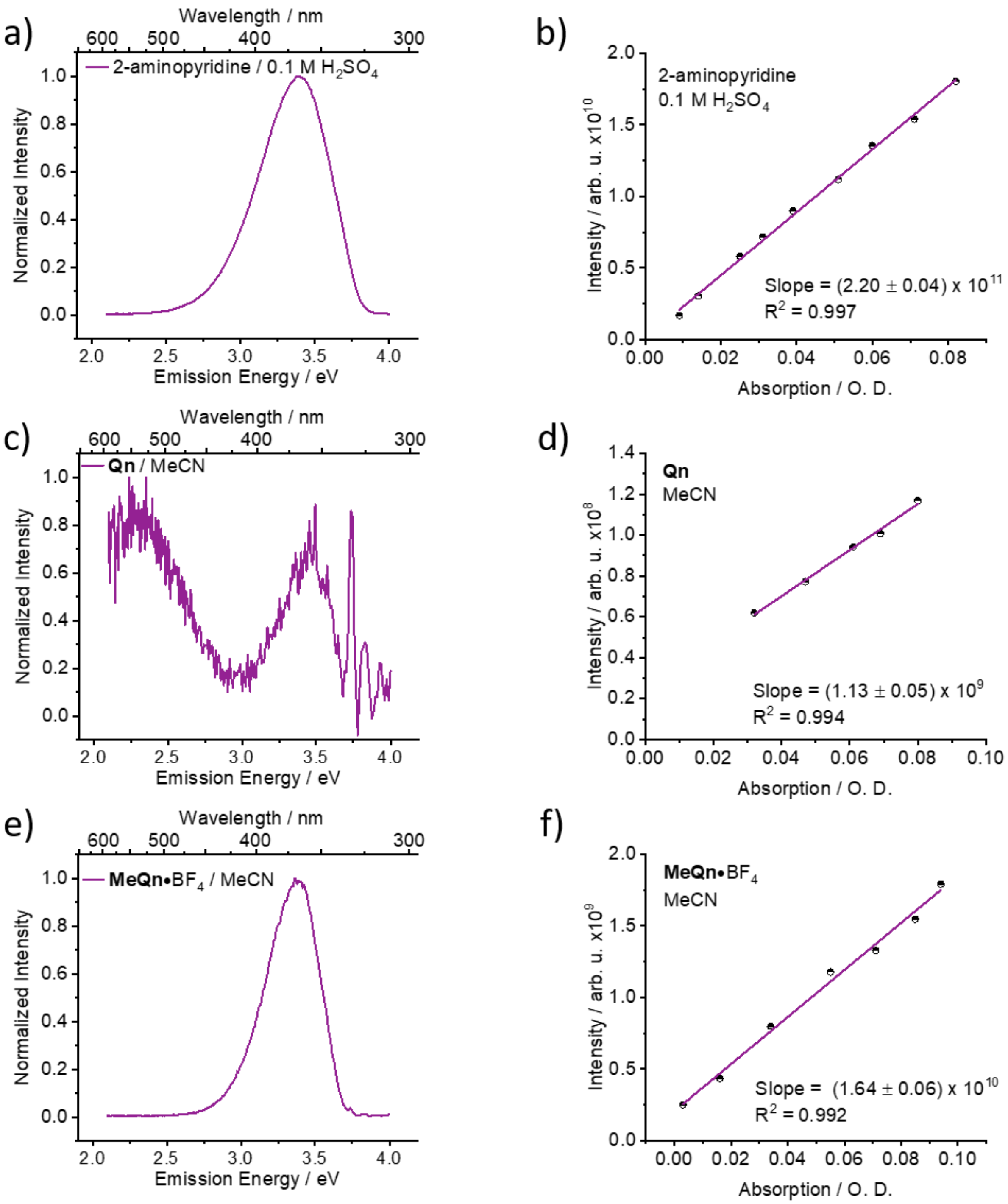

Fig. S22 The emission spectra and emission vs absorption slope of (a-b) 2-aminopyridine in 0.1 M $H_2SO_{4(aq)}$ as a standard, (c-d) **Qn** in MeCN and (e-f) **MeQn**·BF_4 in MeCN.

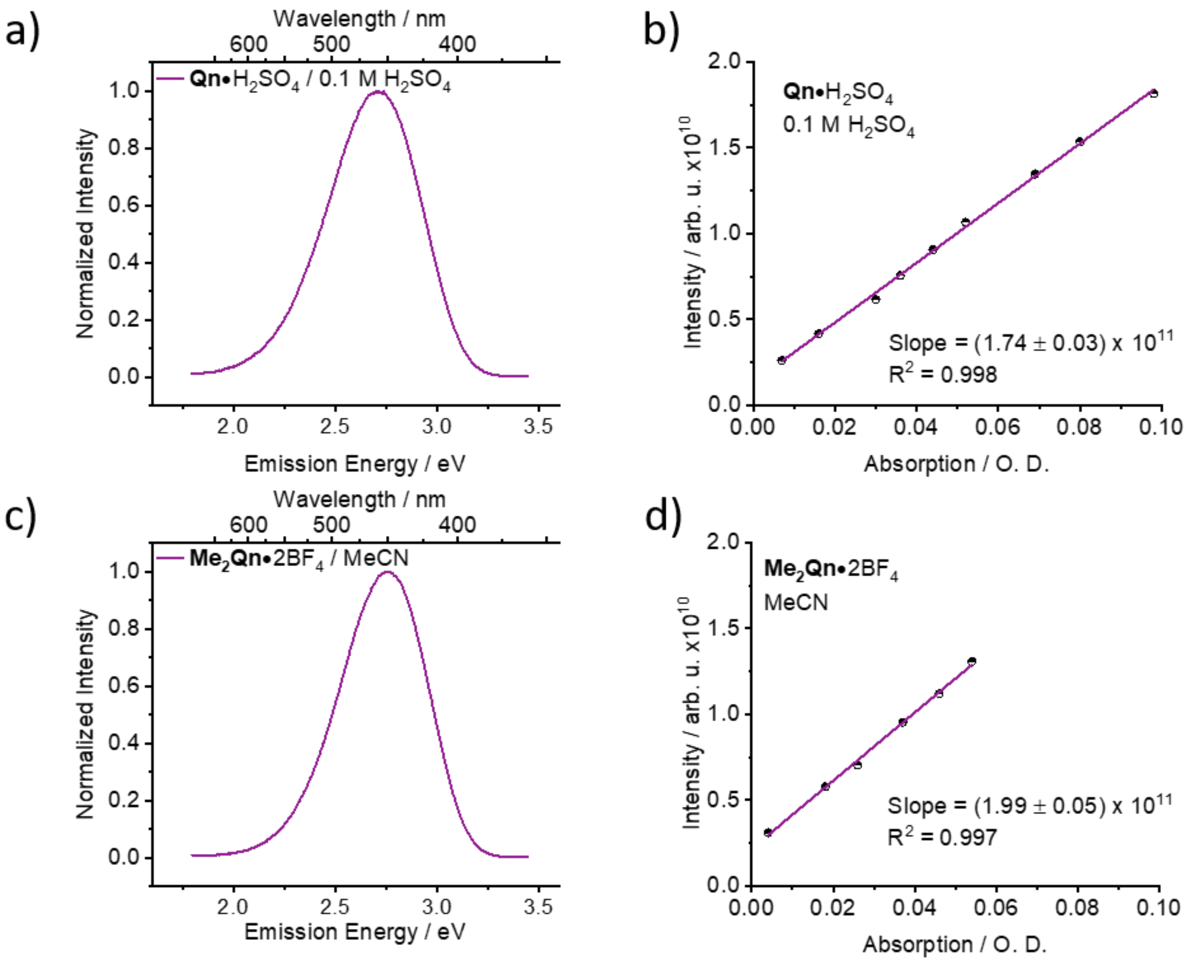

Fig. S23 The emission spectra and emission vs absorption slope of (a-b) **Qn**·H_2SO_4 in 0.1 M $H_2SO_{4(aq)}$ as a standard and (c-d) **Me_2Qn**·$2BF_4$ in MeCN.

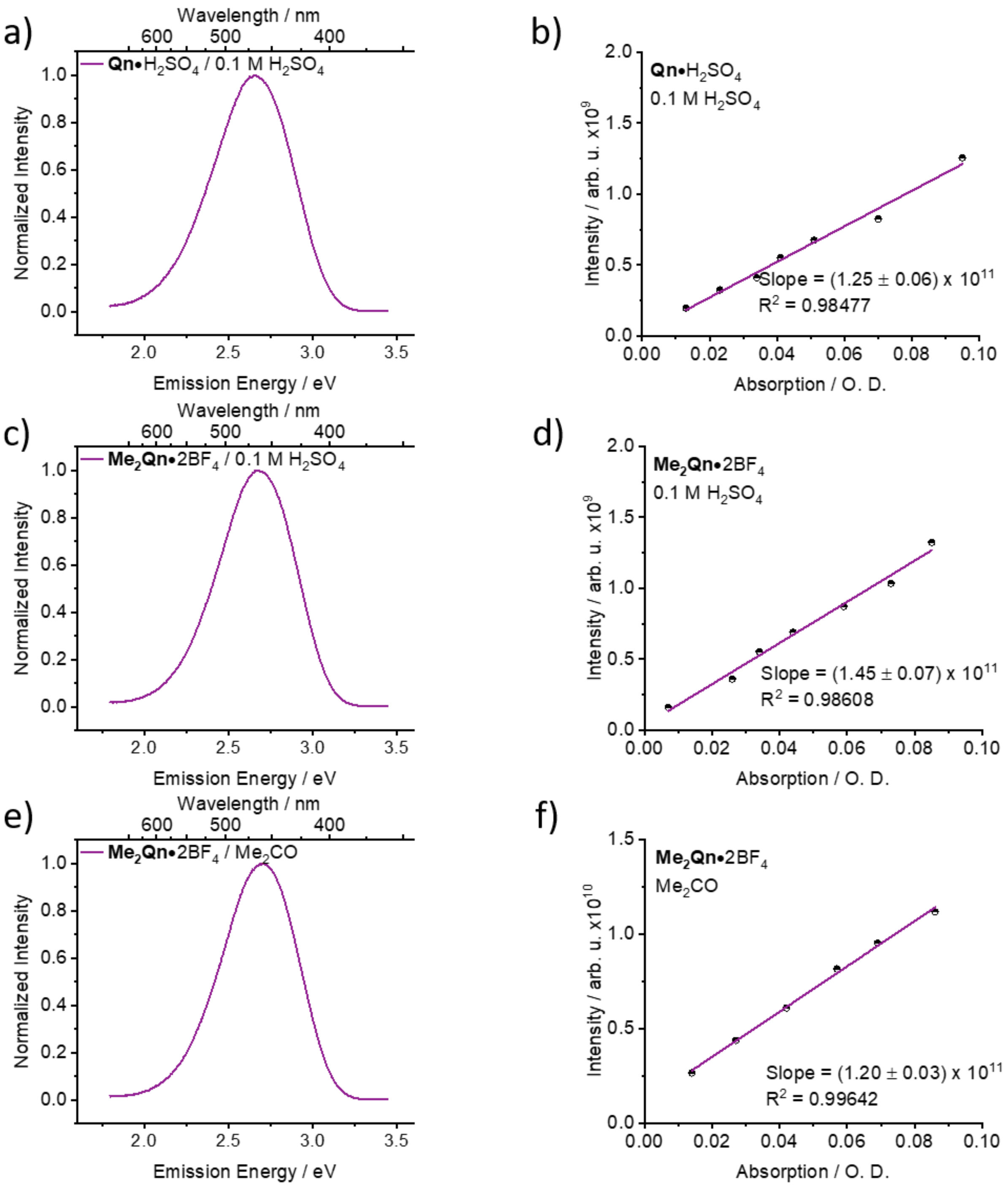

Fig. S24 The emission spectra and emission vs absorption slope of (a-b) **Qn**·H_2SO_4 in 0.1 M $H_2SO_{4(aq)}$ as a standard, (c-d) **Me₂Qn**·$2BF_4$ in 0.1 M H_2SO_4 and (e-f) **Me₂Qn**·$2BF_4$ in acetone (Me_2CO)

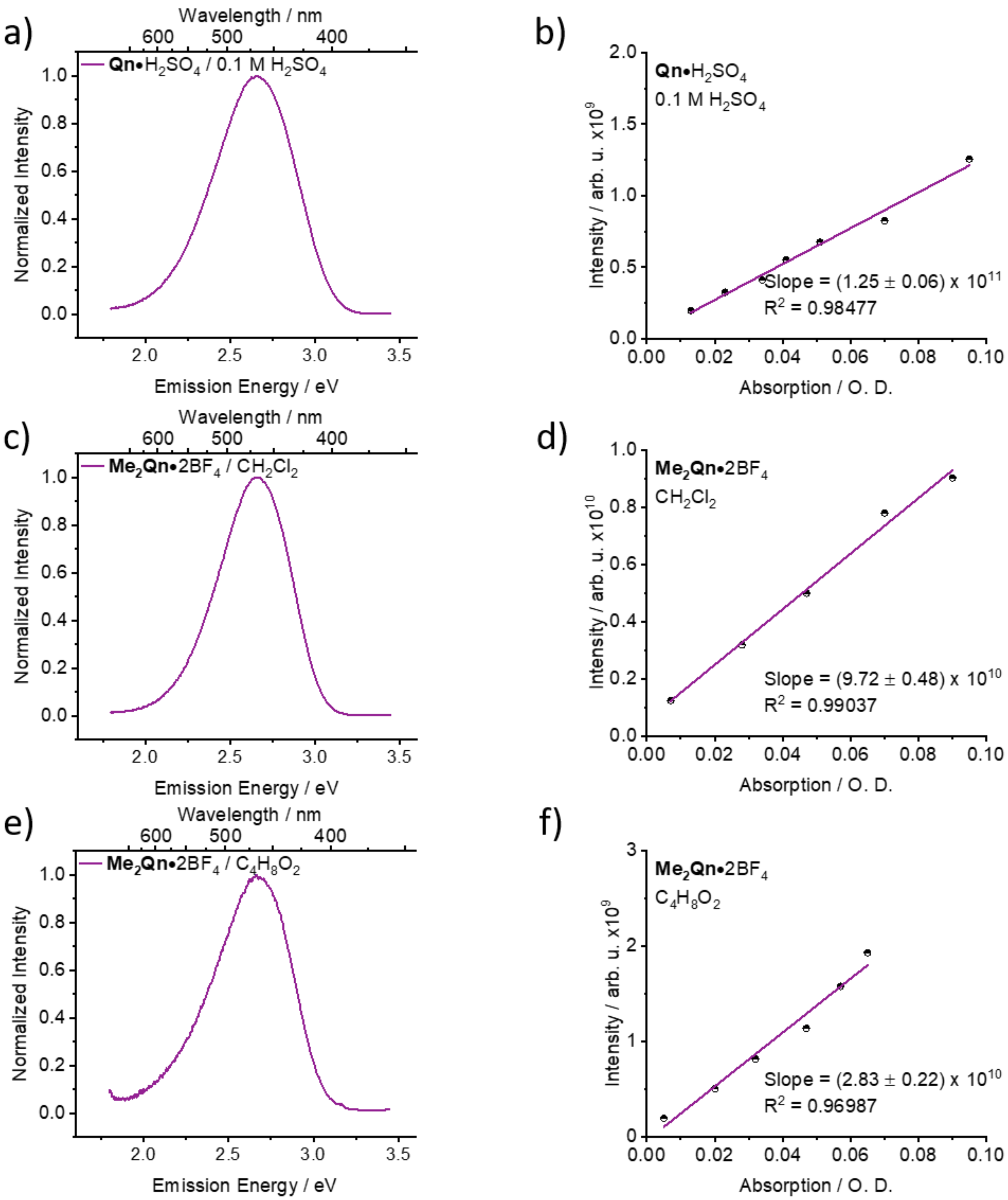

Fig. S25 The emission spectra and emission vs absorption slope of (a-b) **Qn**·H_2SO_4 in 0.1 M $H_2SO_{4(aq)}$ as a standard, (c-d) **Me₂Qn**·$2BF_4$ in dichloromethane (CH_2Cl_2) and (e-f) **Me₂Qn**·$2BF_4$ in ethyl acetate ($C_4H_8O_2$).

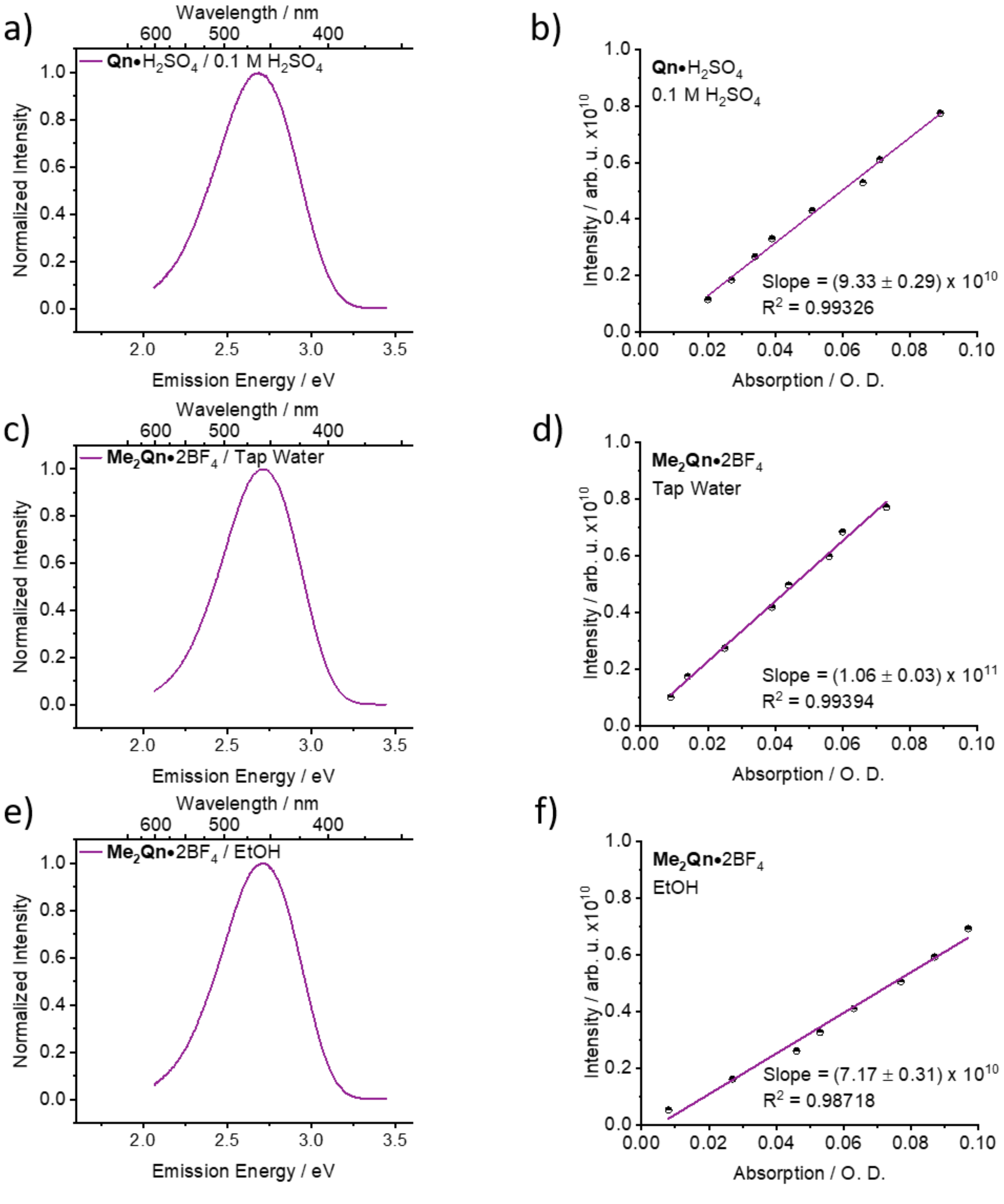

Fig. S26 The emission spectra and emission vs absorption slope of (a-b) **Qn**·H_2SO_4 in 0.1 M $H_2SO_{4(aq)}$ as a standard, (c-d) **Me₂Qn**·$2BF_4$ in tap water and (e-f) **Me₂Qn**·$2BF_4$ in ethanol (EtOH).

Table S2 PLQYs measured for $\mathbf{Me_2Qn}$·$2BF_4$ in an extended series of solvents.

Solvent	Dielectric Constant, ε[9]	Polarity Index[10]	Φ / %
Deionised Water	88	1	70
Tap Water	-	-	62
0.1 M $H_2SO_{4(aq)}$	-	-	62
Acetonitrile (MeCN)	37.5	0.460	63
Ethanol (EtOH)	24.3	0.654	43
Acetone (Me_2CO)	20.7	0.355	54
Dichloromethane (CH_2Cl_2)	9.08	0.309	48
Ethyl Acetate (EtOAc)	6.4	0.228	13

6. Phosphorescence of Qn in a Zeonex Film

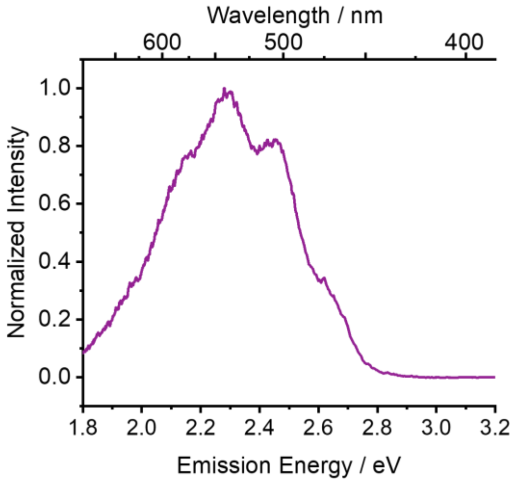

Fig. S27 The phosphorescence emission spectra of a 1 wt% film of **Qn** in zeonex. The film was prepared by dropcasting a PhMe solution and recorded at 40 ms delay and 80 K.

7. Theoretical study

Computational details

Ground-state geometry optimizations of **Qn** and its derivatives were performed both *in vacuo* and using an implicit acetonitrile solvent. We used a protonation of N1 and N2 instead of a methylation, but Table S7 below shows that the effect of such substitution is minimal for emission energies. We employed density functional theory (DFT) with the ωB97X-D exchange-correlation functional[11] and a 6-31G* basis set.[12] This functional contains both long-range and dispersion interaction corrections. Solvent effects were taken into account using self-consistent reaction field (SCRF) model, where the solvent is represented by a dielectric continuum. More specifically, we employed the integral equation formalism of the polarisable continuum model (IEFPCM).[13] Harmonic vibrational frequencies were computed to confirm the nature of all stationary points.

Excited electronic states were computed using linear-response time-dependent density functional theory (LR-TDDFT) at the ωB97X-D/6-31G* level of theory and systematically compared to results obtained with the wavefunction-based second-order algebraic diagrammatic construction method (ADC(2)),[14] used along with a cc-pVDZ basis set.[15] ADC(2) calculations were performed with frozen core and resolution of identity approximations. To justify the use of a small basis set, tests were conducted for comparison with the larger aug-cc-pVDZ basis set.[16]

Vertical transitions were calculated based on the optimized ground-state geometries using LR-TDDFT, with nonequilibrium linear-response IEFPCM solvation (acetonitrile). As our main focus is to determine the emission properties of the studied compounds, we also located minimum energy

structures on the potential energy surface of the first excited state (and also the second one for some specific cases). For these excited-state geometry optimizations, we used LR-TDDFT combined with equilibrium linear-response IEFPCM solvation (acetonitrile). Harmonic vibrational frequencies were computed for excited-state minima. The linear-response approach for implicit solvation models is known to provide reliable excited-state geometries, but less accurate emission energies.[17] Therefore, the emission energies were also computed with the state-specific approach as implemented in Gaussian09. ADC(2) excited-state optimizations were performed only in vacuo, and harmonic frequencies were not computed due to the high computational cost. To estimate the emission energy for the solvated systems, single-point ADC(2) computations with the conductor-like screening model (COSMO)[18] for solvation were performed *a posteriori* on the optimized gas phase geometries. This protocol relies on the assumption that the calculated excited-state structures are similar in gas and solvent phases. We also assume the equilibrium solvation limit, in which the electronic and nuclear degrees of freedom of the solvent adapt to the excited-state (rather than the ground-state) charge distribution of the solute.[19] It is important to note, however, that we do not attempt here to closely reproduce the experimental values with our calculations, but rather to detect the main qualitative changes in absorption and emission upon protonation of quinine.

Excited state characters were analysed by computing natural transition orbitals (NTO),[20] both with TDDFT and ADC(2). All DFT/LR-TDDFT calculations were performed with the Gaussian09 software,[21] while ADC(2) calculations were conducted with Turbomole 7.3.1 program package.[22] Molecular representations were produced with VMD 1.9.2.[23]

Supporting calculations

Quinine can have different conformations, and its stable conformers were analyzed in detail in earlier theoretical and experimental work.[24,25] Here, we have mainly focused our attention on the so called cis-γ-open(3) conformer, which was identified as the most stable conformer based on jet-cooled spectroscopy, vibrational circular dichroism and theoretical calculations.[25] The same conformer is found for the crystal structure of quinine.[26]

The absorption spectrum of quinine contains both locally excited (LE) bright ππ* transition localized on the quinoline moiety, and a higher-lying charge-transfer (CT) state in which the electron is transferred from a *n*(N) orbital of quinuclidine to a π* orbital of quinoline (*n*π*). As shown in Table S3, the LE(ππ*) state is the lowest excited singlet state both with ωB97X-D and ADC(2). At the ground-state (i.e. Franck-Condon) geometry, the CT(*n*π*) state lies higher in energy, and is likely to be overestimated by ωB97X-D. Using a larger basis set does not alter these trends – the relevant transitions are only slightly down shifted.

Table S3 Excited states of **Qn** (*in vacuo*) at the ground-state geometry optimized at ωB97X-D/6-31G* level. Excitation energies are given in eV, along with corresponding oscillator strengths (f).

	ωB97x-D/6-31G*	ωB97x-D/aug-cc-pVDZ	ADC(2)/cc-pVDZ	ADC(2)/aug-cc-pVDZ
S_1	4.47 (LE), f=0.0993	4.35 (LE), f=0.0992	4.27 (LE), f=0.0949	4.15 (LE), f=0.0979
S_2	4.72, f=0.0029	4.67, f=0.0019	4.63, f=0.0024	5.53, f=0.0018
S_3	4.92, f=0.0336	4.81, f=0.0296	4.89 (CT), f=0.0013	4.63 (CT), f=0.0010
S_4	5.44 (CT), f=0.0029	5.34 (CT), f=0.0020	4.97, f=0.0529	4.71, f=0.0061
S_5	5.79, f=0.0002	5.71, f=0.0029	5.51, f=0.0002	4.83, f=0.0400

Upon protonation of the N atoms having an available lone pair, the excited states are either shifted in energy or removed from the spectrum (as they cannot be formed anymore). Table S4 compares the absorption energies of the LE(ππ*) and CT(*n*π*) states of **Qn** and its protonated derivatives, **HQn**$^+$ (proton on N1), **iHQn**$^+$ (proton on N2) and **H**$_2$**Qn**$^{2+}$ (protons on N1 and N2) in acetonitrile solvent. Protonation of N1 prevents the formation of the CT(*n*π*) states, while protonation of N2 causes a large red-shift of the LE(ππ*) and CT(*n*π*) vertical transition (when latter exists). **H**$_2$**Qn**$^{2+}$ exhibits both effects.

Table S4 LE(ππ*) and CT(*n*π*) excited states of **Qn** and its protonated derivatives with ωB97X-D/6-31G* in acetonitrile solvent. Vertical transitions were evaluated at the ground-state geometries optimized at the same level of theory with DFT. Excitation energies are given in eV, along with corresponding oscillator strengths (f).

	**Qn**	**HQn**$^+$	**iHQn**$^+$	**H**$_2$**Qn**$^{2+}$
LE	4.43, f=0.1312	4.38, f=0.1402	3.93, f=0.1289	3.88, f=0.1263
CT	5.32, f=0.0025	-	4.06, f=0.0064	-

The calculated vertical emission energies (Table S5) indicate that protonation of N2 causes a significant red-shift of the emission as compared to the pristine **Qn**. However, the LE emission of **iHQn**$^+$ is expected to be quenched due to the presence of the CT state, i.e., the molecule is likely to undergo nonradiative decay. This is consistent with our experimental findings for the analogous methylated derivatives, which found barely detectable fluorescence for **iMeQn**$^+$. Interestingly, even in **Qn** the CT state falls down in energy upon geometry relaxation, and the nonradiative population exchange between LE and CT states becomes more likely (keeping in mind that CT energies are overestimated by ωB97X-D). Therefore, LE emission in **Qn** has very low intensity, while CT emission becomes almost equally pronounced (see Figure 1 in the main text). Protonation of N1 causes only

slight red-shift in emission. Again, **H_2Qn^{2+}** benefits from two effects; the colour of its fluorescence is altered, while deleterious nonradiative effects are less likely due to the lack of CT state.

Table S5 Vertical emission energies computed with ωB97X-D/6-31G* and ADC(2)/cc-pVDZ. Note that with ωB97X-D equilibrium linear-response solvation (acetonitrile) is used, while ADC(2) optimizations were performed in gas phase. Nevertheless, the qualitative conclusions are similar. Emission energies are given in eV, along with corresponding oscillator strengths (f).

		**Qn**	**HQn^+**	**$iHQn^+$**	**H_2Qn^{2+}**
ωB97x-D	LE	3.81 (S_1), f=0.2641	3.76 (S_1), f=0.2635	converge to CT	3.21 (S_1), f=0.1741
	CT	3.20 (S_1), f=0.0291	-	2.20 (S_1), f=0.0074	-
ADC(2)	LE	3.75 (S_1), f=0.1078	3.59 (S_1), f=0.1262	2.69 (S_2; above CT), f=0.0777	2.58 (S_1), f=0.0924
	CT	2.44 (S_1), f=0.0071	-	1.01 (S_1), f=0.0019	-

We have also evaluated emission energies based on a state-specific solvation schemes (Table S6), in which solvent is supposed to fully equilibrate with the excited state from the solute, while the ground state of the solute is treated in a non-equilibrium limit. The underlying assumption is that the excited-state lifetime is sufficiently long such that the slow and fast degrees of freedom of the solvent have sufficient time to adapt.[19] One possible explanation for the too low emission energies of the CT states could be related to a breakdown of this assumption, the CT states being too short lived to allow for a full relaxation of the solvent (leading to an overestimation of relaxation effects in the calculations). This effect is particularly striking for **Qn**, which exhibits experimentally an emission band centred at around 2.2 eV, see Figure 1). We note here that such underestimation of charge transfer bands with state-specific implicit solvent models were reported in the literature.[27,28] Explicit solvent effects might be required to improve the description of this band. The LE states of **Qn** and **$iHQn^+$** may also not have sufficiently long lifetime due to the nonradiative effects mentioned above, but the variations in LE emission energy with and without state-specific solvation are generally not very large.

Table S6 Emission energies from state-specific equilibrium solvation (acetonitrile). The optimised geometries are the same as those in Table S5, but additional single-point computations were conducted. Emission energies are given in eV.

		Qn	HQn^+	$iHQn^+$	H_2Qn^{2+}
ωB97x-D	LE	3.75	3.69	-	3.00
	CT	1.41	-	1.01	-
ADC(2)	LE	3.61	3.47	2.56	2.43
	CT	1.46	-	0.36	-

Finally, Table S7 shows that methylation has almost the same impact on the calculated fluorescence as protonation.

Table S7 Comparison of vertical emission energies for the protonated and methylated **Qn** derivatives. Excited states were optimised with ωB97X-D/6-31G* and equilibrium linear-response solvation (acetonitrile). Emission energies are given in eV, along with corresponding oscillator strengths (f).

	HQn^+	$iHQn^+$	H_2Qn^{2+}
LE	3.76 (S_1), f=0.2635	converge to CT	3.21 (S_1), f=0.1741
CT	-	2.20 (S_1), f=0.0074	-
	**$MeQn^+$**	**$iMeQn^+$**	**Me_2Qn^{2+}**
LE	3.75 (S_1), f=0.2574	converge to CT	3.23 (S_1), f=0.1840
CT	-	2.23 (S_1), f=0.0088	-

7. References

1 A. T. R. Williams, S. A. Winfield and J. N. Miller, *Analyst*, 1983, **108**, 1067.

2 W. H. Melhuish, *J. Phys. Chem.*, 1961, **65**, 229–235.

3 R. Rusakowicz and A. C. Testa, *J. Phys. Chem.*, 1968, **72**, 2680–2681.

4 O. V. Dolomanov, L. J. Bourhis, R. J. Gildea, J. A. K. Howard and H. Puschmann, *J. Appl. Crystallogr.*, 2009, **42**, 339–341.

5 G. M. Sheldrick, *Acta Crystallogr. Sect. A Found. Crystallogr.*, 2008, **64**, 112–122.

6 H. D. Flack, *Acta Crystallogr. Sect. A Found. Crystallogr.*, 1983, **39**, 876–881.

7 R. W. W. Hooft, L. H. Straver and A. L. Spek, *J. Appl. Crystallogr.*, 2008, **41**, 96–103.

8 M. Kubicki, T. Borowiak, K. Gawrońska and J. Gawroński, *Zeitschrift fur Naturforsch. - Sect. B J. Chem. Sci.*, 2000, **55**, 1083–1088.

9 Dixon Valve Dielectric Constants, https://www.dixonvalve.com/sites/default/files/product/files/brochures-literature/dielectric-constant-values.pdf, (accessed 24 February 2020).

10 C. Reichardt, *Solvents and Solvent Effects in Organic Chemistry*, Wiley, 2002.

11 J.-D. Chai and M. Head-Gordon, *Phys. Chem. Chem. Phys.*, 2008, **10**, 6615.

12 R. Krishnan, J. S. Binkley, R. Seeger and J. A. Pople, *J. Chem. Phys.*, 1980, **72**, 650–654.

13 J. Tomasi, B. Mennucci and R. Cammi, *Chem. Rev.*, 2005, **105**, 2999–3093.

14 A. Dreuw and M. Wormit, *Wiley Interdiscip. Rev. Comput. Mol. Sci.*, 2015, **5**, 82–95.

15 T. H. Dunning, *J. Chem. Phys.*, 1989, **90**, 1007–1023.

16 R. A. Kendall, T. H. Dunning and R. J. Harrison, *J. Chem. Phys.*, 1992, **96**, 6796–6806.

17 R. Improta, G. Scalmani, M. J. Frisch and V. Barone, *J. Chem. Phys.*, 2007, **127**, 074504.

18 A. Klamt and G. Schüürmann, *J. Chem. Soc. Perkin Trans. 2*, 1993, 799–805.

19 B. Lunkenheimer and A. Köhn, *J. Chem. Theory Comput.*, 2013, **9**, 977–994.

20 R. L. Martin, *J. Chem. Phys.*, 2003, **118**, 4775–4777.

21 M. J. Frisch, G. W. Trucks, H. B. Schlegel, G. E. Scuseria, M. A. Robb, J. R. Cheeseman, G. Scalmani, V. Barone, G. A. Petersson, H. Nakatsuji, X. Li, M. Caricato, A. Marenich, J. Bloino, B. G. Janesko, R. Gomperts, B. Mennucci, H. P. Hratchian, J. V. Ortiz, A. F. Izmaylov, J. L. Sonnenberg, D. Williams-Young, F. Ding, F. Lipparini, F. Egidi, J. Goings, B. Peng, A. Petrone, T. Henderson, D. Ranasinghe, V. G. Zakrzewski, J. Gao, N. Rega, G. Zheng, W. Liang, M. Hada, M. Ehara, K. Toyota, R. Fukuda, J. Hasegawa, M. Ishida, T. Nakajima, Y. Honda, O. Kitao, H. Nakai, T. Vreven, K. Throssell, J. A. Montgomery Jr, J. E. Peralta, F. Ogliaro, M. Bearpark, J. J. Heyd, E. Brothers, K. N. Kudin, V. N. Staroverov, T. Keith, R. Kobayashi, J. Normand, K. Raghavachari, A. Rendell, J. C. Burant, S. S. Iyengar, J. Tomasi, M. Cossi, J. M. Millam, M. Klene, C. Adamo, R. Cammi, J. W. Ochterski, R. L. Martin, K. Morokuma, O. Farkas, J. B. Foresman and D. J. Fox, *Gaussian, Inc., Wallingford CT*, 2016.

22 F. Furche, R. Ahlrichs, C. Hättig, W. Klopper, M. Sierka and F. Weigend, *Wiley Interdiscip. Rev. Comput. Mol. Sci.*, 2014, **4**, 91–100.

23 W. Humphrey, A. Dalke and K. Schulten, *J. Mol. Graph.*, 1996, **14**, 33–38.

24 M. K. Bilonda, L. Mammino and S. Scheiner, *Molecules*, 2017, **22**, 1–13.

25 A. Sen, A. Bouchet, V. Lepère, K. Le Barbu-Debus, D. Scuderi, F. Piuzzi and A. Zehnacker-Rentien, *J. Phys. Chem. A*, 2012, **116**, 8334–8344.

26 B. Pniewska and A. Suszko-Purzycka, *Acta Crystallogr. Sect. C Cryst. Struct. Commun.*, 1989, **45**, 638–642.

27 P. Kautny, F. Glöcklhofer, T. Kader, J. M. Mewes, B. Stöger, J. Fröhlich, D. Lumpi and F. Plasser, *Phys. Chem. Chem. Phys.*, 2017, **19**, 18055–18067.

28 J. M. Mewes, J. M. Herbert and A. Dreuw, *Phys. Chem. Chem. Phys.*, 2017, **19**, 1644–1654.
